# Repeat to gene expression ratios in leukemic blast cells can stratify risk prediction in acute myeloid leukemia

**DOI:** 10.1186/s12920-021-01003-z

**Published:** 2021-06-26

**Authors:** M. Onishi-Seebacher, G. Erikson, Z. Sawitzki, D. Ryan, G. Greve, M. Lübbert, T. Jenuwein

**Affiliations:** 1 Max Planck Institute of Immunobiology and Epigenetics, Freiburg, Germany; 2 Faculty of Biology, International Max Planck Research School for Molecular and Cellular Biology (IMPRS-MCB) and University of Freiburg, Freiburg, Germany; 3 Department of Medicine I, Medical Center – University of Freiburg, Faculty of Medicine, University of Freiburg, Freiburg, Germany; 4 German Cancer Consortium (DKTK), Freiburg, Germany; 5 Present Address: Novartis Institute for Biomedical Research (NIBR), Basel, Switzerland; 6 Centre for Discovery Brain Sciences, The University of Edinburgh, Edinburgh, UK

## Abstract

**Background:**

Repeat elements constitute a large proportion of the human genome and recent evidence indicates that repeat element expression has functional roles in both physiological and pathological states. Specifically for cancer, transcription of endogenous retrotransposons is often suppressed to attenuate an anti-tumor immune response, whereas aberrant expression of heterochromatin-derived satellite RNA has been identified as a tumor driver. These insights demonstrate separate functions for the dysregulation of distinct repeat subclasses in either the attenuation or progression of human solid tumors. For hematopoietic malignancies, such as Acute Myeloid Leukemia (AML), only very few studies on the expression/dysregulation of repeat elements were done.

**Methods:**

To study the expression of repeat elements in AML, we performed total-RNA sequencing of healthy CD34 + cells and of leukemic blast cells from primary AML patient material. We also developed an integrative bioinformatic approach that can quantify the expression of repeat transcripts from all repeat subclasses (SINE/ALU, LINE, ERV and satellites) in relation to the expression of gene and other non-repeat transcripts (i.e. R/G ratio). This novel approach can be used as an instructive signature for repeat element expression and has been extended to the analysis of poly(A)-RNA sequencing datasets from Blueprint and TCGA consortia that together comprise 120 AML patient samples.

**Results:**

We identified that repeat element expression is generally down-regulated during hematopoietic differentiation and that relative changes in repeat to gene expression can stratify risk prediction of AML patients and correlate with overall survival probabilities. A high R/G ratio identifies AML patient subgroups with a favorable prognosis, whereas a low R/G ratio is prevalent in AML patient subgroups with a poor prognosis.

**Conclusions:**

We developed an integrative bioinformatic approach that defines a general model for the analysis of repeat element dysregulation in physiological and pathological development. We find that changes in repeat to gene expression (i.e. R/G ratios) correlate with hematopoietic differentiation and can sub-stratify AML patients into low-risk and high-risk subgroups. Thus, the definition of a R/G ratio can serve as a valuable biomarker for AML and could also provide insights into differential patient response to epigenetic drug treatment.

**Supplementary Information:**

The online version contains supplementary material available at 10.1186/s12920-021-01003-z.

## Background

Repeat elements constitute around 50% of the mammalian genome and, in addition to their role as insertional mutagens, have been involved in functions for genome evolution and stability [34, 42], embryonic development [22, 31, 43], immune response [12] and in fine-tuning of gene regulatory networks [17, 45, 51]. While the majority of repeat elements are permutated and silent, it has been estimated that around 10–15% maintain transcriptional competence [7, 53]. In addition, several subclasses of repeat elements have also been shown to be deregulated in cancer [8]. Derepression of centromeric satellite repeats (ALR, GSAT, HSATII), for example, have been found in multiple solid human cancers [57, 66]. Deregulated satellite RNA can induce repeat expansion at pericentric heterochromatin [4], generate genomic instabilities [66, 67] and aberrantly sequester some key epigenetic factors, such as YBX1 [35] or the Polycomb complex PRC1 [28]. By contrast, therapeutic activation of endogenous retroviruses (ERV) has been found to trigger an innate immune response in human prostate and breast cancer cell lines [11, 47], suggesting that some types of cancer maintain a suppressed level of ERV expression in order to evade immune surveillance. The analysis of repeat element expression/dysregulation has recently been extended to several hematological malignancies, particularly Acute Myeloid Leukemia [13, 14].

Acute Myeloid Leukemia (AML) is a heterogeneous cancer of the myeloid lineage of blood cells, which, in addition to prevalent genetic lesions, also exhibit epigenetic alterations in DNA methylation and histone modifications [63, 65]. Treatments against AML with ‘epigenetic drugs’, such as the DNMT inhibitors Azacytidine and Decitabine [41], or combination therapies with DNMTi and ATRA [26] or DNMTi and HDACi [5] are effective and are under clinical investigation. However, the mechanism behind these epigenetic alterations and how these epigenetic drugs target leukemic cells are only now starting to become apparent. In addition, no clear biomarkers that can predict AML treatment response have yet been identified. Therefore, we set out to determine repeat element expression in AML, by high-throughput sequencing of total-RNA of AML patient samples compared to healthy blood controls. The repeat expression analysis was further extended to poly(A)-RNA sequencing data sets of AML patient samples from the Blueprint [10] and TCGA consortia [9]. Our integrative analysis indicates that repeat element expression is dynamically regulated during hematopoiesis and that differences in repeat to gene expression ratios associate with distinct subgroups of AML patients and correlate with overall prognosis. Through this approach, we identified repeat to gene expression ratios (R/G ratios) as a signature in AML that may serve as a prognostic biomarker through which epigenetic drug efficacy may also be tested.

## Methods

### Purification of primary human CD34 + cells

Unused human blood transfusions were obtained from the University of Freiburg Medical Center (Transfusionsmedizin des Universitätsklinikums Freiburg). 50 ml blood was diluted 1:1 in PBS and separated on a Ficoll gradient (Pancoll, Ibian Technologies). The interphase (buffy coat) was transferred to a 50 ml Falcon tube, the cell suspension was washed in PBS and cells were processed for MACS sorting which was done with anti-CD34 MicroBeads (Miltenyibiotec). Around 0.5–1 × 10^6^ CD34 + cells are typically obtained from one buffy coat. Cells were resuspended in 1 ml Trizol (Sigma) and kept at − 80 °C until RNA extraction.

Samples of pre-therapeutic and newly diagnosed AML patients were obtained from the Hematology, Oncology and Stem cell Transplantation department of the University of Freiburg, Medical Center. Leukemic blast cells were isolated from peripheral blood of 7 patients and only for one patient were the leukemic blast cells isolated from bone marrow. For 6 patients, both bone marrow and peripheral blood blast percentages were analyzed (in the remaining 2 patients, no bone marrow puncture could be performed), indicating that the median bone marrow and blood blast percentages are highly comparable (61% and 59%) (see Additional file 11: Table 1). Leukemic blast cells were purified by MACS sorting with both anti-CD34 and anti-CD117 MicroBeads (Miltenyibiotec).

### Isolation of total RNA from primary human CD34 + cells

Total RNA from around 0.5–1 × 10^6^ cells was isolated with Trizol (Sigma), digested with TurboDNAse (Ambion), washed and resuspended in H_2_O. The RNA integrity was confirmed with an RNA Bioanalyzer (Agilent).

### HiSeq RNA sequencing of non-poly(A) selected total RNA

Comparable amounts (250 ng–1 ug) of total RNA was converted into non-poly(A) selected, ribosomal RNA depleted (TruSeq RNA Library Prep Kit v2) cDNA libraries following Illumina protocols. The cDNA libraries were sequenced on a NextSeq 500 (Illumina) or HiSeq 2500 (Illumina) platform using a 75 bp paired-end approach to give a coverage of 26–78 million reads per sample.

### Blueprint data sets

In the Blueprint-HSC dataset [10], an ultra-low RNA input kit (Clontech SMARTer Ultra Low RNA Kit) for library preparation was used [52], which may also enable the detection of repeat element transcripts that are only weakly poly-adenylated. The Blueprint-HSC cDNA libraries were done in an unstranded manner using 100 bp paired-end reads (100 bp paired–end reads also for Blueprint-MPP and 75 bp paired-end reads for Blueprint-CMP) and had a coverage of 42–103 million reads per sample. The Blueprint-AML dataset enriched for poly(A)-RNA using standard mRNA library generation protocols that can analyze transcripts in a strand-specific manner. The Blueprint-AML libraries used 75 bp paired-end reads and had a coverage of 53–79 million reads per sample. In accordance with the Ft Lauderdale agreement, these data are available for additional analysis under https://europepmc.org/articles/PMC6363099.

### TCGA data sets

For the TCGA-AML dataset, mRNA purification by oligo-dT was supplemented by the use of random hexamer primers (TCGA-AML NEJM). The TCGA-AML libraries used 50 bp paired-end reads and had a coverage of 21–34 million reads per sample. Data were downloaded from the documentation published for the TCGA-AML study [9].

### Bioinformatic analysis of repeat element expression and R/G ratio

Paired-end reads in Fastq files were first trimmed (stringency 2) using Trim Galore! (v0.4.0) and aligned to the human genome build GRChg38 from Ensembl using STAR (Dobin et al. [16]) with the “–outFilterMultimapNmax 100 –winAnchorMultimapNmax 100” options. Short sequencing reads (75–100 bp) containing repeat element sequences align to multiple positions in the genome and only the minority of these reads can be uniquely assigned to a distinct location. In our bioinformatic pipeline, we therefore defined the total number of repeat transcripts for each repeat subclass, rather than analyzing repeat transcripts at individual genomic loci. We also did not filter according to intergenic and genic (i.e. intronic) repeat assignments. This maximized the number of read counts and also enabled a comparative analysis of the total number of reads for each distinct repeat subclass and for genes. Repeat and gene expression was quantified with TETranscripts version 2.00 [32]. Repeat transcripts refer to annotated repeat elements obtained from the TETranscripts website and non-repeat transcripts refer to annotated genes from Gencode (release 24). The resulting counts were then analyzed in R with the DESeq2 (Love et al. [40]) package to obtain normalized expression levels for repeat elements and genes, using rlog transformation for the Uniklinik Freiburg samples and the variance stabilizing transformation function for the Blueprint and TCGA samples. These transformations stabilize the mean–variance relationship seen in all RNA-seq expression datasets and were done as detailed in Love et al. ([40]). *P*-values were extracted using the DESeq2 package (Love et al. [40]), calculated by the Wald test and adjusted using the BH (Benjamini-Hochberg) method.

An R/G ratio is defined by the median of normalized counts for all repeat transcripts versus the median of normalized counts for all gene transcripts. For example, a R/G ratio of 0.82 had a median rlog normalized expression for repeat transcripts of 7.83 and a median rlog normalized expression for gene transcripts of 9.57. Data visualization was performed using ggplot2 [29, 62]. Heatmaps for dysregulated repeat element expression in Uniklinik Freiburg and Blueprint datasets were generated using pheatmap in R (pheatmap: Pretty heatmaps. R package version 1.0.8. https://CRAN.Rproject.org/package=pheatmap).

### MA plots

Differential expression analysis for the Uniklinik Freiburg samples was conducted with DESeq2, using gender, batch and cell type as the design matrix. Differential expression analysis of TCGA patient samples based on R/G ratio stratification used gender and R/G ratio stratification group as the design matrix. MA plots were created using the baseMean values on the x-axis and the log2 fold change values on the y-axis (DESeq2), using ggplot2 for visualization. Multiple testing adjustments were done using the BH (Benjamini-Hochberg) method.

### Inter-patient variation plots

Normalized counts (DESeq2) for each patient were plotted along the x-axis using consistent patient ordering. The median of the normalized counts per repeat was plotted as a black horizontal line.

### Consensus coverage plots for repeat sequences

For coverage plots, reads were aligned to consensus repeat sequences rather than the human genome. Consensus repeat sequences were first downloaded from Repbase [2] and then highly similar (sequence identity > 95%) sequences merged together using CD-HIT version 4.6 (Fu et al. [23]). After alignment, per-base coverage normalized to average 1X coverage was determined using bamCoverage from DeepTools version 2.5.3 (Ramírez et al. [44]). Final coverage tracks were then plotted in R using the Gviz package (Hahne and Ivanek 2016).

### Stratification of AML patient samples by R/G ratio

The R/G ratios were calculated as the median of repeat element expression divided by the median of gene expression per patient sample. Patient samples were stratified based on z-score normalized R/G ratios using the cut_interval function from ggplot2, to create patient subgroups with equal R/G ratio range. For our study, the numeric vector represents the z-score normalized R/G ratios and n represents the “high-repeat”, “mid-repeat” and “low-repeat” categories (therefore, n was set to 3). We did not use a cut-point in between the categories, as using the cut_interval function is a more unbiased way to divide numeric data into categorical data. The Uniklinik Freiburg low-repeat AML samples had R/G ratios between 0.82 and 0.85 and the Uniklinik Freiburg high-repeat AML samples had R/G ratios between 1.03 and 1.08. The Blueprint low-repeat AML samples had R/G ratios between 0.97 and 1.01 and the Blueprint high-repeat AML samples had R/G ratios between 1.14 and 1.17. The TCGA low-repeat AML samples had R/G ratios between 0.98 and 1.09 and the TCGA high-repeat AML samples had R/G ratios between 1.21 and 1.32.

### Gene ontology annotations and ingenuity pathway analysis

The downstream functional analyses were generated through the use of IPA (Qiagen Inc, https://www.qiagenbioinformatics.com/products/ingenuity-pathway-analysis). IPA is a frequently updated and comprehensive database that uses GSEA-like statistics. The log2FoldChange values from the differential expression analysis were used, after filtering for a minimum expression (baseMean expression > 100 normalized reads), fold-change (absolute value of log2FoldChange > 1), and significance (adjusted *p*-value < 0.05) as inputs for IPA. This filtering strategy extracts statistically significant expression changes that are meaningfully above background level. The data were exported and plotted using R/Bioconductor.

### Survival probability

Survival analysis was conducted on AML patient samples using metadata available from TCGA. Covariates used were the R/G ratio-based groups, age, gender, and AML cytogenics risk category. Univariate Cox proportional hazards regression modeling to determine the effect of covariates on survival was performed using the R survival package (Therneau ([58]). A Package for Survival Analysis in S version 2.38, < URL: https://CRAN.R-project.org/package=survival >), which showed R/G ratio-based groups, age, and cytogenetic risk category to have statistically significant regression coefficients. Plots were created with the survminer library (Kassambara and Kosinski [33]). survminer: Drawing Survival Curves using ‘ggplot2’. R package version 0.4.0. https://CRAN.R-project.org/package=survminer). Multivariate statistical modeling using the Cox proportional hazards model was performed using R/G ratio-based groups, age, gender, AML subtypes and acute myeloid leukemia cytogenetic risk category as covariates, where the R/G ratio-based groups and cytogenetics risk category remained statistically significant. To check for the proportional-hazards assumption and to confirm a Cox regression, we used the cox.zph R function from the survival package (Therneau [58]). The forest plot for Cox proportional hazards multivariate analysis was generated using ‘ggforest’ function from survminer library.

### Correlation between R/G ratios and chromatin factor expression

The Pearson correlation coefficient (PCC) between R/G ratios and chromatin factor expression levels (rlog for Uniklinik Freiburg AML and vst for Blueprint AML and TCGA AML) were calculated using the cor() function in R for the Uniklink Freiburg, the Blueprint-AML, and the TCGA-LAML dataset separately. *P*-values were adjusted using the BH (Benjamini-Hochberg) method. The mean PCCs are shown on the plots.

## Results

### Dysregulation of satellite and LTR/ERV repeats in AML patient samples

Repeat elements and repetitive DNA constitute around 50% of the human genome (Additional file 1: Figure S1A and S1B) and have been classified as Long Interspersed Nuclear Elements (LINE) (~ 22%), Short Interspersed Nuclear Elements (SINE), also called ALU (*Arthrobacter luteus* restriction endonuclease) (~ 13%), and Endogenous RetroViruses (ERV) with their associated regulatory elements within the Long Terminal Repeats (LTR) (~ 9%). Satellite repeats are subdivided into centromeric (ALR for alpha like repeats, GSAT and HSATII) and pericentric (BSR for beta satellite repeats and HSTAIII) repeats and make up around 4% of the human genome [24, 53]. These distinct subclasses of repeat elements greatly differ in size and repeat organization (Additional file 1: Figure S1C) and by their potential to impart transcription regulatory sequences (Additional file 2: Figure S2).

From the Hematology, Oncology and Stem Cell Transplantation department of the University of Freiburg Medical Center (Uniklinik Freiburg), we obtained purified leukemic blast cells (see "Methods" section) from eight pre-therapeutic and newly diagnosed AML patients (see Additional file 11: Table 1). Leukemic blast cells were isolated from peripheral blood of 7 patients and only for one patient were the leukemic blast cells isolated from bone marrow. Although the range of leukemic blasts in the blood versus bone marrow can greatly differ in AML tumor dynamics, peripheral blood blast or bone marrow blast isolates have been shown to be highly comparable for the analysis of diagnostic markers (morphology, immunocytochemistry and immunophenotype) [61]. In addition to these leukemic samples, we also obtained buffy coats from five healthy blood transfusion donors (see "Methods" section). From the buffy coats, CD34 + cells were FACS sorted, but not further sub-purified and represent a mixed population of progenitor and more differentiated myeloid cells. From these cell samples, we prepared total RNA sequencing libraries and performed paired-end, 75 bp Hiseq RNA sequencing with the number of mapped reads ranging from ~ 20 to 75 million reads (median of ~ 42 million reads) (see "Methods" section). The isolation of total RNA, rather than selection for poly(A) RNA, should ensure that we obtain a more complete expression profile of repeat elements that also includes solo-LTR transcripts, truncated LINE transcripts and all satellite repeat sequences.

AML patient samples can be subtyped into distinct FAB groups [3], of which we obtained material classified as M1 (n = 2), M2 (n = 3), and M4 (n = 3) (Additional file 11: Table 1). The M1 subtype refers to leukemic myeloblast cells with minimal differentiation, the M2 subtype to myeloblasts with some maturation, while the M4 subtype represents a state of myelomonocytic differentiation.

**Fig. 1:**
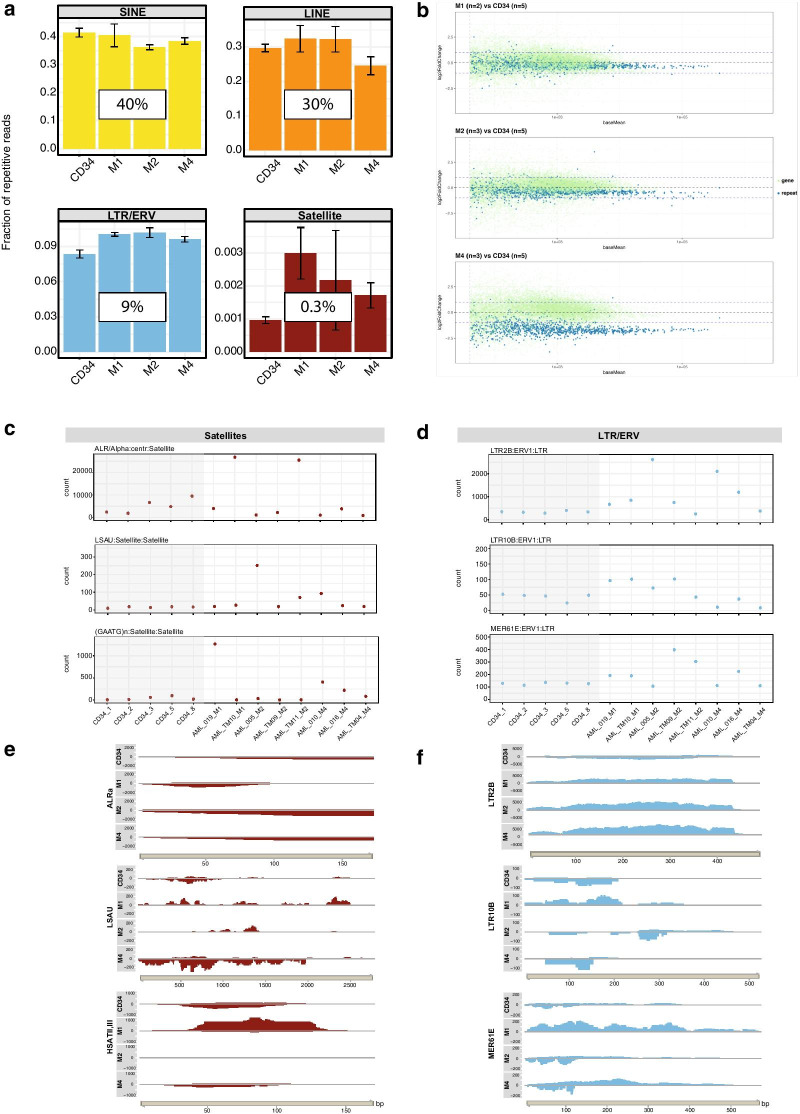
Repeat element expression in AML patient samples (Uniklinik Freiburg). **a** Fraction of repeat transcripts that align to the main repeat classes in the CD34 + control and in the M1, M2 and M4 AML patient samples (Uniklinik Freiburg). 40% of reads consist of SINE/ALU repeats, around 30% are LINE elements, 9% LTR/ERV, and between 0.1 and 0.3% are Satellite repeats. Samples are grouped into CD34 + control and the M1, M2 and M4 AML subtypes. Error bars represent the standard error of the mean (SEM). **b** MA-plots (mean expression versus the log2 fold change) depicting changes in repeat expression in the in the CD34 + controls (n = 5) and the three AML subtypes: M1 (n = 2), M2 (n = 3) and M4 (n = 3). X-axis indicates the baseMean values and y-axis the log2 fold change values. **c**/**d** Inter-patient variation plots show normalized read counts for distinct repeat types in Satellite repeats (**c**) or LTR/ERV elements (**d**). Each data point represents one sample for each of the CD34 + control (n = 5) and AML samples (n = 8). **e**/**f** Coverage plots represent transcript reads that are aligned to consensus repeat sequence for distinct repeat types in Satellite repeats (**e**) or LTR/ERV elements (**f**). Per-base coverage is normalized to average 1X coverage as determined using bamCoverage from DeepTools

We first determined the overall expression of repeat elements by comparing the fraction of repeat reads that align to the main repeat subclasses. Around 40% of all repeat reads comprise SINE/ALU repeats, around 30% contain LINE elements, ca. 9% LTR/ERV, and only between 0.1 and 0.3% are satellites (Fig. 1a). While the fraction of repeat reads between the CD34 + controls and the distinct AML subtypes did not consistently change for SINE/ALU and LINE repeats, we observed a modest increase for LTR/ERV reads in all three AML subtypes. Although their overall expression is very low, satellite repeats were most derepressed in the AML subtypes. Statistical inference for differential expression was done by the Wald test and p-values were adjusted using the BH (Benjamini-Hochberg) method. Unless directly specified (see below), these differences have a non-significant tendency.

We next used an MA plot (mean expression versus the log2 fold change) to identify dysregulation of distinct repeat elements in the CD34 + controls and the three AML subtypes (Fig. 1b). For the M1, M2 and M4 AML subtypes, the MA plots show a general down-regulation for a large number of repeat elements in the M2 (19 significantly down-regulated repeats; adj. *p*-value < 0.05, log2FC <  − 1) and, most pronounced, in the M4 (922 significantly down-regulated repeats; adj. *p*-value < 0.05, log2FC <  − 1) AML subtype. This global repression of repeat elements is not reflected by overall changes in gene expression, as the average level of gene transcripts remains constant in the M1, M2 and M4 AML subtypes. Despite this general down-regulation, there are several satellite (e.g. ALR, LSAU, HSATII,III) and LTR/ERV (e.g. LTR2B ERV1, LTR10B ERV1, MER61E ERV1) repeats that appear enriched or upregulated in at least two of the AML subtypes (Additional file 3: Figure S3).

We then analyzed inter-patient variation by plotting normalized read counts for each of these repeat elements and per AML patient sample (Fig. 1c/d). Centromeric ALR repeat transcripts, which are also derepressed in other cancers [57], are found elevated in 2/8 AML patient samples. The LSAU (Long Sau3a) satellite sequence refers to a ~ 2.8 kb long, GC-rich repeat sequence that is interspersed among beta satellite arrays [2]. LSAU transcripts are derepressed in 1/8 AML patient samples. The pericentric satellite sequences (GAATG)n and (CATTC)n (HSATII,III) are also overexpressed in 1/8 AML patient samples (Fig. 1c). Since overexpression occurs in four distinct M1 and M2 AML patient samples (there is no overlap for dysregulation of a distinct satellite subtype in the different AML patient samples), these data indicate that 50% (4/8) of AML patients display deregulation of satellite repeats. For the LTR/ERV repeat class, LTR2B, which functions as the regulatory element for the ERV type Harlequin-int, is overexpressed in 3/8 AML patient samples. LTR10B, as well as its associated HERVIP10B3-int, is overexpressed in 4/8 AML patient samples. MER61E is an LTR for MER4-type ERV and is derepessed in 3/8 AML patient samples (Fig. 1d). Together, 88% (7/8) of distinct AML patient samples display elevated transcript levels of at least one of these LTR repeat elements.

To further illustrate derepression of these selected examples, we generated coverage plots (see "Methods" section), where the number of repeat reads from one CD34 + sample and from individual AML patients were aligned to the consensus sequence of a distinct repeat type that was derived from the repeat element library of RepBase (Bao W., et al. 2015). The coverage plots were done in a strand-specific manner and are shown for the ALRalpha, LSAU and HSATII,III satellite repeats (Fig. 1e). For the LTR/ERV repeats, coverage plots are displayed for LTR2B ERV1, LTR10B ERV1 and MER61E ERV1 (Fig. 1f and data not shown). Together, this combined bioinformatic analysis in AML patient samples from the Uniklinik Freiburg indicates that we can detect dysregulation of particular satellite and LTR/ERV repeat types. However, there is considerable inter-patient variation and the low number of AML patient samples does not reveal a robust signature for a distinct repeat type that would consistently be derepressed in AML patient samples vs. CD34 + healthy control samples.

### Repeat element expression in Blueprint data sets

To expand our analysis, we also gained access to RNA sequencing data of healthy CD34 + and AML patient samples that were generated within the Blueprint consortium [10]. In contrast to our Hiseq RNA sequencing of total RNA preparations with CD34 + cells and AML patient samples from the Uniklink Freiburg, the Blueprint data used poly(A)-RNA selected libraries (see "Methods" section). Also, with its focus on the epigenomic profiling of human blood cells and the analysis of hematopoietic differentiation, the Blueprint samples for healthy CD34 + cells had been subdivided into hematopoietic stem cells (HSC, n = 3), multipotent progenitor cells (MPP, n = 3), and common myeloid progenitor cells (CMP, n = 3). In addition to these 9 CD34 + controls, we also used RNA sequencing data from 14 AML patient samples that were, however, not subtyped based on the FAB classification.

**Fig. 2:**
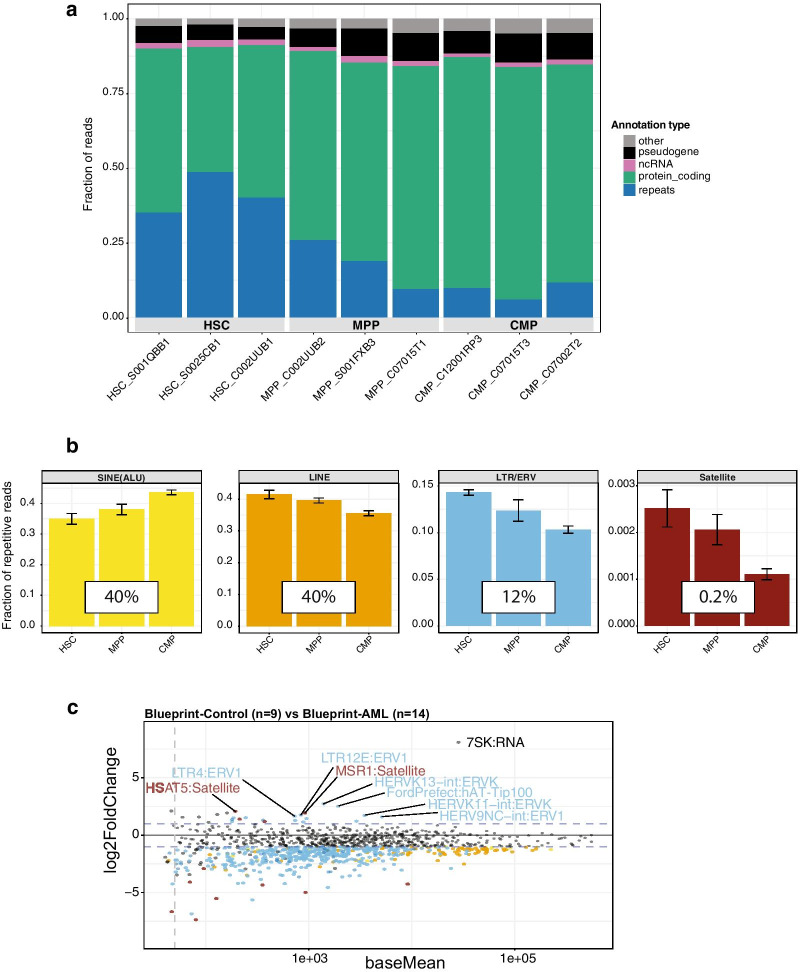
Repeat element expression in Blueprint AML data sets. **a** Fraction of repeat transcripts (blue) versus protein coding transcripts (green) in the HSC (n = 3), MPP (n = 3) and CMP (n = 3) CD34 + cell populations. **b** Fraction of repeat transcripts for distinct repeat classes in HSC, MPP and CMP samples. Error bars represent the standard of the mean (SEM). **c** MA-plots (mean expression versus the log2 fold change) comparing CD34 + control (n = 9) versus AML patient samples (n = 14). Dots above and below the dashed lines are statistically significant as confirmed by multiple testing adjustments using the BH (Benjamini–Hochberg) method. Some examples for de-repressed Satellite repeats and LTR/ERV elements are highlighted. Red dots represent Satellite repeats, blue dots LTR/ERV elements and orange dots LINE repeats

We first analyzed overall repeat expression (i.e. the sum of all repeat reads for all distinct repeat classes) in the different subpopulations of healthy CD34 + cells. We then compared the fraction of repeat transcripts with the fraction of gene and other unique (as annotated in Gencode) transcripts from within the entire set of read counts. The data show that there is progressive down-regulation of repeat expression when cells proceed in their differentiation, such that HSC have a high number of repeat reads, MPP have a reduced number and CMP have the lowest number of repeat reads (Fig. 2a). This relative expression of repeat vs. gene transcripts also establishes a repeat to gene expression ratio (‘R/G ratio’) that may be useful for the comparative analysis of genomic expression profiles in stem/progenitor cells as compared to more differentiated cells.

We next determined the fraction of repeat reads that align to the main repeat subclasses. Around 40% of all repeat reads comprise SINE/ALU repeats, around 40% contain LINE elements, ca. 12% LTR/ERV, and only around 0.2% are satellites (Fig. 2b). While the fraction of repeat reads for SINE/ALU transcripts modestly increase from HSC to MPP to CMP, we detect a progressive decrease for LINE and LTR/ERV transcripts and for satellite repeats (Fig. 2b), although these data have a non-significant tendency.

We then used an MA plot to analyze differential expression of distinct repeat elements between the 9 CD34 + controls and the 14 Blueprint AML patient samples (Fig. 2c). Similar, but not as pronounced as with the data analysis of AML patient samples from the Uniklinik Freiburg (see Fig. 2b), we observe a partial down-regulation for a large amount of repeat elements. There are, on the other hand, several satellite (e.g. HSAT5, adj. *p*-value = 0.04 and MSR1, adj. *p*-value = 0.00015) and LTR/ERV (e.g. LTR12E ERV1, adj. *p*-value = 2.046e−07; and HERVK13-int ERVK, adj. *p*-value = 4.72e−05) repeat transcripts that are appreciably enriched in the Blueprint AML patient samples (Fig. 2c). We also generated heatmaps for the top 30 dysregulated repeat elements in the Blueprint and Uniklinik Freiburg data sets. There is no common repeat subtype that is up-regulated in these top 30 hits of the Blueprint or Uniklinik Freiburg AML samples and only the D20S16 satellite is found to be down-regulated in both data groups (Additional file 4: Figure S4).

### Repeat element expression in TCGA AML data sets

We next obtained access to RNA sequencing data of AML patient samples that were generated within The Cancer Genome Atlas (TCGA) initiative [9]. Similar to the Blueprint data sets, the TCGA used poly(A)-RNA selected libraries (see "Methods" section). In total, we processed raw Hiseq RNA sequencing data from 98 TCGA AML patient samples. The selected TCGA AML patient samples had been subtyped based on the FAB classification as M1 (n = 35), M2 (n = 35) and M4 (n = 28).

To gain insight into inter-patient variation, we determined normalized read counts for distinct repeat elements within each of the main repeat classes (Additional file 5: Figure S5, Additional file 6: Figure S6 and data not shown) in every individual TCGA patient sample. For satellite repeats, we analyzed 22 diverse satellite sequences, of which 15 had a mean expression above cutoff (baseMean expression > 100 normalized reads) (see Additional file 5: Figure S5). We used this cutoff to extract statistically significant expression changes that are meaningfully above background level. Of these 15 satellite sequences, 7 (SATR2, SATR1, MSR1, REP522, HSAT5, LSAU and GSATII) showed, to a varying degree, read counts that deviate by two–threefold above a median expression level, as it was determined for the TCGA data sets (see "Methods" section). For LTR/ERV repeats, we analyzed 578 diverse subtypes, of which 549 had a mean expression above cutoff (baseMean expression > 100 normalized reads). In particular, we focused on 11 differentially expressed LTR elements that drive ERV-1, ERV-K and ERV-L retrotransposons (see Additional File 6: Figure S6). Using similar analyses with ‘inter-patient variation plots’ for 171 LINE subtypes and for 57 SINE/ALU repeats did not robustly expose a distinct LINE or SINE/ALU element that would considerably be derepressed in AML patient samples (data not shown).

Thus, we again observed considerable heterogeneity for satellite and LTR/ERV repeat expression and high inter-patient variation.

### Repeat to gene expression ratios can stratify AML patient subgroups

**Fig. 3:**
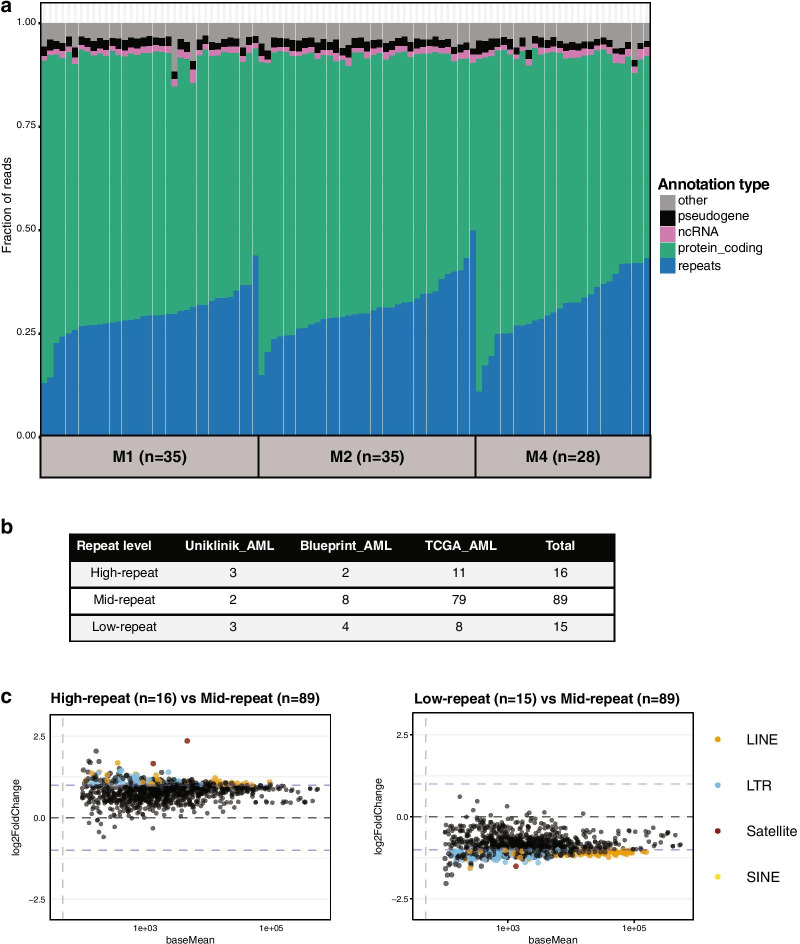
R/G ratios can stratify AML patient subgroups. **a** Fraction of repeat transcripts (blue) versus fraction of protein coding transcripts (green) in TCGA AML patient samples. Within each M1 (n = 35), M2 (n = 35) and M4 (n = 28) AML subtype, patient samples are ordered according to increasing fraction of repeat transcripts. **b** Table showing the number of patient samples in each cohort (Uniklinik Freiburg AML, Blueprint AML and TCGA AML) that were classified by R/G ratio in High-repeat, Mid-repeat and Low-repeat subgroups. **c** MA-plots (mean expression versus the log2 fold change) depicting changes in repeat expression in the High-repeat (n = 16) versus Mid-repeat (n = 89) and in the Low-repeat (n = 15) versus Mid-repeat (n = 89) AML patient samples. x-axis indicates the baseMean values and y-axis the log2 fold change values (DESeq2). Dots above and below the dashed lines are statistically significant as confirmed by multiple testing adjustments using the BH (Benjamini–Hochberg) method

We therefore were seeking to derive a more general definition for repeat element dysregulation that would more appropriately identify a repeat expression signature in human AML. This notion was further supported by our earlier observations on the global down-regulation of repeat element expression in the M4 AML subgroup from the Uniklinik Freiburg (see Fig. 1b) and during the progression of the Blueprint HSC to the Blueprint CMP cells (see Fig. 2a). We quantified overall repeat expression in relation to genes and other unique transcripts in every AML patient sample of the TCGA M1 (n = 35), TCGA M2 (n = 35) and TCGA M4 (n = 28) AML cohorts. The profiles reveal that within each of the three TCGA AML subtypes, there are two distinct AML patient subgroups, which either have a considerably reduced or elevated fraction of repeat transcripts (Fig. 3a).

We then determined the R/G ratios for the TCGA AML patient samples (n = 98), the Blueprint AML patient samples (n = 14) and the Uniklinik Freiburg AML patient samples (n = 8). The R/G ratio model can sub-stratify 16 AML patient samples (11 TCGA AML, 2 Blueprint AML and 3 Uniklinik Freiburg AML) with a high R/G ratio (‘high-repeat’) and 15 AML patient samples (8 TCGA AML, 4 Blueprint AML and 3 Uniklinik Freiburg AML) with a low R/G ratio (‘low-repeat’) (Fig. 3b) (see "Methods" section). In addition, we could subgroup 89 AML patient samples with an intermediate R/G ratio (‘mid-repeat’) that we used as a reference to determine differential expression of repeat elements in the ‘high-repeat’ and the ‘low-repeat’ AML patient samples. Notably, this sub-stratification by R/G ratios is now independent of FAB classification or other cytogenetic (chromosomal aberrations) or gene mutation based parameters and only relies on the relative expression of repeat versus non-repeat transcripts. While there are differences in HiSeq RNA library preparations and variability in the sequencing depths (see "Methods" section), all three data groups (Uniklinik Freiburg, Blueprint and TCGA) allow for a comparable coverage and detection of repeat element transcripts and gene transcripts (Additional file 7: Figure S7).

The MA plot (Fig. 3c) validates that repeat elements are broadly derepressed in the ‘high-repeat’ AML patient samples (n = 16) compared to the ‘mid-repeat’ AML patient samples (n = 89) and that ‘low-repeat’ AML patient samples (n = 15) have a general down-regulation of repeat elements. ‘Inter-patient variation plots’ for these sub-stratified ‘low-repeat’, mid-repeat’ and ‘high-repeat’ AML patient samples further indicate that all repeat classes (satellites, LTR/ERV, LINE and SINE/ALU elements) equally segregate with R/G ratios, as defined by the cut_interval function (see Additional file 8: Figure S8).

### ‘High-repeat’ and ‘low-repeat’ AML patient subgroups have distinct alterations of cancer and inflammation pathways

**Fig. 4:**
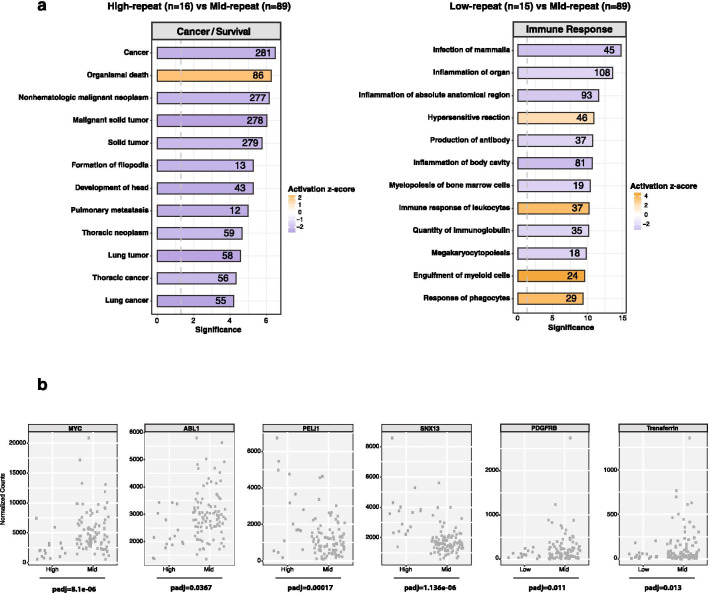
IPA analysis in AML patient subgroups with high or low R/G ratios. **a** Ingenuity pathway analysis (IPA) performed in High-repeat (n = 16) versus Mid-repeat (n = 89) and in Low-repeat (n = 15) versus Mid-repeat (n = 89) AML patient samples. As inputs for the IPA, the log2FoldChange values from the differential gene expression analysis were used, after filtering for a minimum expression (baseMean expression > 100 normalized reads), fold-change (absolute value of log2FoldChange > 1), and significance (adjusted *p*-value < 0.05). z-score values are indicated by color code, where purple represents down-regulation and orange up-regulation of a pathway. The numbers specify the number of genes that are dysregulated in each of the different pathways. **b** Example for specific genes that are dysregulated in High-repeat (High) versus Mid-repeat (Mid) (*MYC, ABL1, PELI1, SNX13*) or in Low-repeat (Low) versus Mid-repeat (Mid) (*PDGFR, TRANSFERRIN*) AML patient samples. Shown are normalized read counts for gene specific transcripts. Statistical significance is indicated by adjusted *p*-values

In addition to up- and down-regulation of repeat transcripts, we also analyzed differential gene expression between the ‘high-repeat’ vs. ‘mid-repeat’ AML patient samples and between the ‘low-repeat’ versus ‘mid-repeat’ AML patient samples. We identified Ingenuity Pathway Analysis (IPA, Qiagen) predicted downstream pathways that are either activated or repressed in the ‘high-repeat’ or ‘low-repeat’ AML patient samples compared to ‘mid-repeat’ AML patient samples (Fig. 4a). In the ‘high-repeat’ AML patient samples, we found the repression of cancer pathways (including metastasis and neoplasms) and the activation of a death/apoptosis pathway. We identify, for example, down-regulation of *MYC* and *ABL1* oncogenes and increased expression of *PELLINO* and a sorting nexin *SNX13* (Fig. 4b, left and middle panels). By contrast, the ‘low-repeat’ AML patient samples showed changes in immune response-related pathways, such that hypersensitive reaction pathways are activated, whereas infection and inflammation pathways are suppressed. We found, for example, that expression of *PDGF receptor B* and *Transferrin* is decreased (Fig. 4b, right panels). These distinct differences of altered gene expression pathways between ‘high-repeat’ and ‘low-repeat’ AML patient samples are *in addition* to upregulated Toll-like receptor signaling, NF-kb activation and interferon signaling, which are similarly stimulated in both ‘high-repeat’ and ‘low-repeat’ AML patient subgroups as compared to the CD34 + (Blueprint) control cells (see Additional file 9: Figure S9).

### Increased survival probability for AML patients with a high R/G ratio

**Fig. 5:**
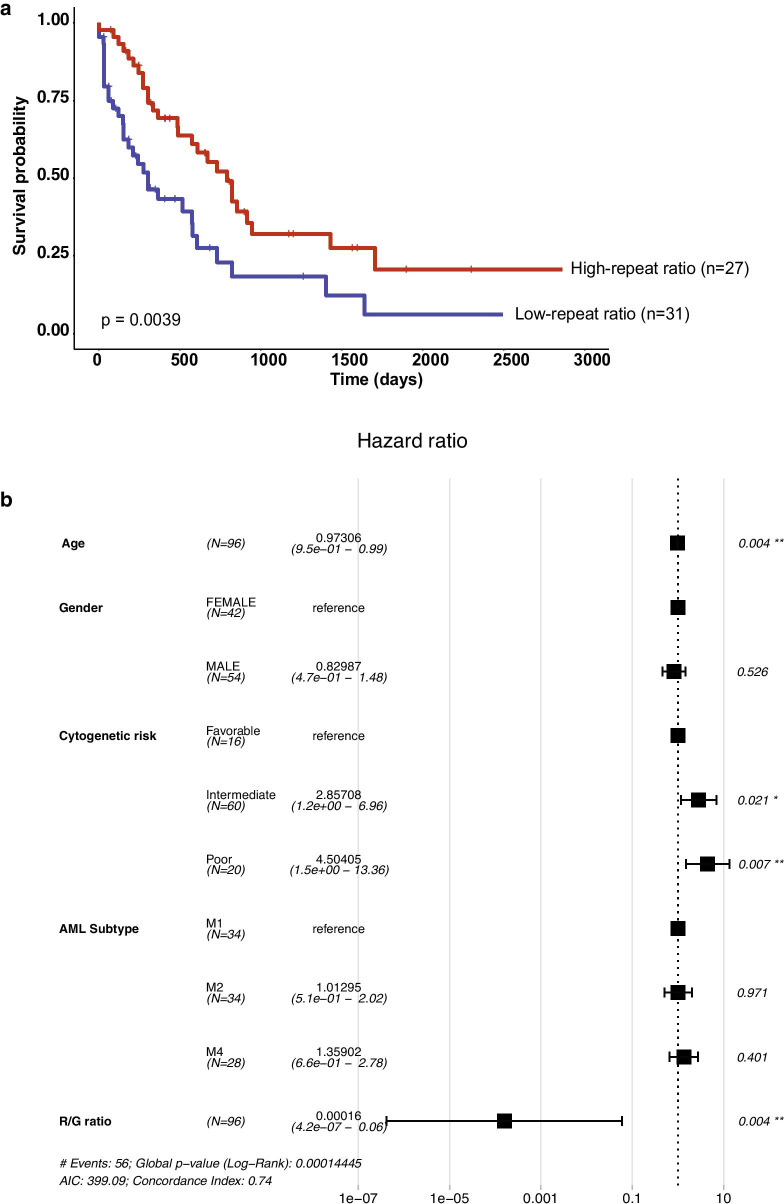
Survival probability and Cox hazard risks in AML patient subgroups with high or low R/G ratios. **a** Kaplan–Meier univariate survival analysis was performed on AML patient samples using metadata available from the TCGA study. Kaplan–Meier univariate survival analysis found the High-repeat TCGA AML patients (n = 27) had a higher overall survival probability as compared to the Low-repeat TCGA AML patients (n = 31) (*p*-value of 0.0039). **b** Cox proportional hazards multivariate analysis was performed on AML patient samples using metadata available from the TCGA study by comparing age, gender, cytogenetic risk category (favorable, intermediate and adverse), AML subtypes and R/G ratios. All *p*-values < 0.05 are considered significant. A hazard ratio above 1 (dashed line) indicates an increased risk for an event (relapse, progression, death), while a hazard ratio below 1 indicates a reduced risk. R/G ratios significantly segregate from age and cytogenetic risk categories with a hazard index of 0.00016 (95% confidence interval: 4.2e − 07–0.06; *p*-value of 0.004)

The IPA data suggested that the stratification of AML patient samples by the R/G ratio model could possibly be used as a prognostic biomarker in the risk prediction for AML. Patient data were available from the entire TCGA AML cohort (98 patient samples), of which 58 TCGA AML patient samples (across the TCGA M1, TCGA M2 and TCGA M4 subtyping) had an event (i.e. relapse, progression or death). We determined the R/G ratio of these 58 TCGA AML patient samples which could define two AML patient subgroups with either a high R/G ratio or a low R/G ratio. We then performed a Kaplan–Meier univariate survival analysis and found that the ‘high-repeat’ TCGA AML patients (n = 27) had a higher overall survival probability as compared to the ‘low-repeat’ TCGA AML patients (n = 31) (*p*-value of 0.0039) (Fig. 5a). We also used a Cox proportional hazards multivariate analysis on 96 patient samples from the TCGA AML cohort (2 patient samples had no identified cytogenetic risk). This Cox multivariate hazard analysis compared age, gender, cytogenetic risk category (favorable, intermediate and adverse), AML subtypes and R/G ratios. It showed that R/G ratios significantly segregate from age and cytogenetic risk categories with a median hazard index of 0.00016 (95% confidence interval: 4.2e−07–0.06; *p*-value of 0.004) (Fig. 5b). The R/G based stratification of AML subgroups is therefore independent of age and cytogenetic risk classification, although R/G ratios have a broad range of reduced hazard risk. We conclude that the R/G ratio model can serve as a novel biomarker in the stratification of AML patient samples and may also be useful in the prognosis of high-risk vs. low-risk AML patients.

### Expression of chromatin factors that correlate with high or low R/G ratio in the AML patient subgroups

A variety of chromatin-modifying mechanisms (e.g. DNA methylation, histone modification and nucleosome remodeling) have been involved in the regulation of repeat elements, where a repressed chromatin state silences their expression during normal development and differentiation. To address whether distinct chromatin-modifying enzymes or other chromatin factors are also differentially dysregulated in the AML patient subgroups with a high or low R/G ratio, we interrogated ~ 100 instructive chromatin enzymes/factors (see Additional file 12: Table 2) with a particular focus on components that have been shown to regulate repeat element expression.

**Fig. 6:**
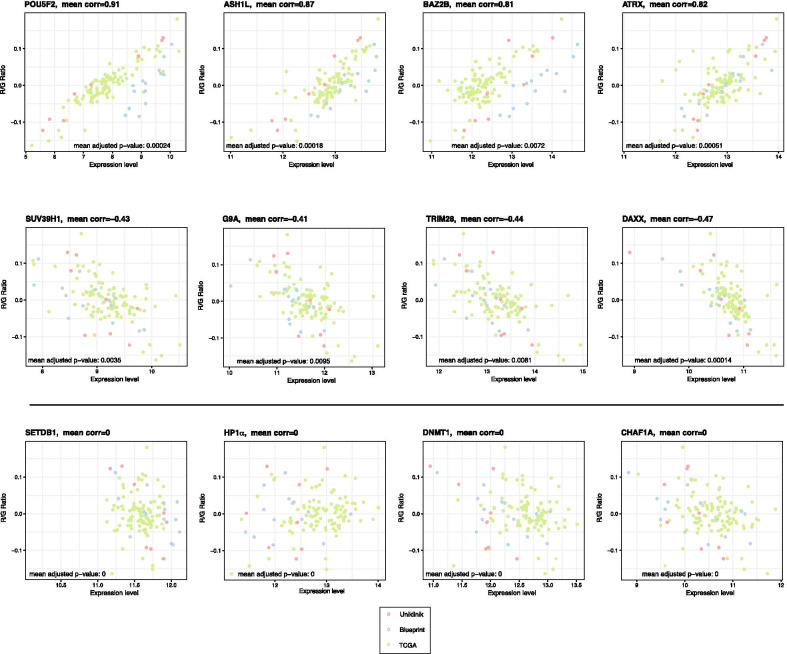
Expression of chromatin factors in AML patient samples with high or low R/G ratios. Pearson correlation coefficients between the R/G ratio and chromatin enzyme/factor expression in each individual AML patient sample from the High-repeat (n = 16), Mid-repeat (n = 89) and Low-repeat (n = 15) subgroups, as they were classified in Fig. 3b. A high correlation (mean PCC > 0.8) is found for POU5F2, ASH1L, BAZ2B and ATRX, a medium correlation (mean PCC > 0.4) is found for SUV39H1, G9A, TRIM28 and DAXX and no correlation (mean PCC = 0) is found for SETDB1, HP1α, DNMT1 and CHAF1A

We calculated the Pearson correlation coefficients between the R/G ratio and chromatin enzyme/factor expression in each individual AML patient sample from the ‘high-repeat’, ‘mid-repeat’ and ‘low-repeat’ data sets. A high correlation (mean corr > 0.8) was revealed for transcription factors (e.g. ZNF407, POU5F2), ‘activating’ histone modifying enzymes (e.g. KDM3A/JMJD1, KMT2C/MLL3 and ASH1L) and chromatin remodelers (e.g. ATRX) (Fig. 6, top panels). The transcription factor POU5F2 is a paralog of Oct4 [56]. Increased expression of POU5F2 (x-axis) correlates with higher R/G ratios (y-axis). Another example is ASH1L, a histone lysine methyltransferase (KMT) that methylates histone H3 lysine 36 (H3K36) [25], often in synergy with another activating KMT, Mixed-Lineage Leukemia 3 (KMT2C/MLL3) that methylates H3K4 [54]. BAZ2B (Bromo-domain Adjacent to Zinc finger domain 2B), which binds to acetylated histone H3 lysine 14 (H3K14Ac) [21] and the chromatin remodeler ATRX (Alpha Thalassemia/mental Retardation syndrome X) [38] also correlate with elevated R/G ratios.

We also found, although with only a weaker correlation (mean corr > 0.4), described repeat element repressors that display increasing expression in AML patient samples with a low-level repeat transcription (Fig. 6, middle panels). For example, the SUV39H1 [7] and G9A [55] KMT, which methylate H3K9, anti-correlate with higher R/G ratios. TRIM28 (KAP1), a transcriptional co-repressor that is recruited to ERV repeat elements through KRAB-Zinc finger transcription factors [48, 59], is also expressed at higher levels in AML patient samples with low R/G ratios, as is the histone H3.3 chaperone DAXX (death domain associated protein). Recently, it has been shown that DAXX association with SETDB1-TRIM28 (KAP1) selectively represses ERV repeat elements [19]. Notably, our analysis indicates an inverse correlation profile for DAXX and ATRX, although a DAXX-ATRX complex has been reported to be targeted to tandemly-repeated telomeric and centromeric sequences [38, 49]. It is possible that in the AML patient samples where ATRX expression is high (and DAXX expression is low), uncomplexed ATRX may function as an activating component solely through its nucleosome remodeling activity [30]. Finally, we did not find an apparent correlation profile for other chromatin enzymes/factors that have been shown to repress repeat element expression (Fig. 6, bottom panels). Expression levels for the H3K9 KMT SETDB1, the heterochromatin protein HP1α, the DNA methyltransferase DNMT1 and the chromatin assembly factor CHAF1 [64] did not correlate with R/G ratios in the AML patient samples. A recent screen in several human AML cancer cell lines has identified up-regulated SETDB1 to secure silencing of retrotransposons, thereby attenuating an anti-cancer immune and interferon response [15].

## Discussion

With the advanced technologies for high-throughput RNA sequencing and the growing interest in non-coding RNA, it has become more and more apparent that repeat RNA transcripts have functional roles both during normal and perturbed development and that they also contribute to the adaptation of cell fates [20, 22, 31, 43]. Specifically for cancer, aberrant expression of heterochromatin-derived satellite RNA has been identified as a novel hallmark in a variety of human solid tumors [57, 66] and forced expression of satellite repeat transcripts has recently been shown to induce breast neoplasia in a mouse model [67]. Overexpression of human HSATII,III repeats has also been detected as circulating RNA in the blood of pancreatic cancer patients, suggesting that aberrant satellite repeat expression can serve as a diagnostic marker of disease [36, 37]. Together, these data indicate derepression of satellite repeat transcription to be a tumor driver. By contrast, cancer cells appear to suppress transcription of LTR/ERV elements and of other retrotransposons, in order to attenuate an anti-tumor immune response. Therapeutic activation of LTR/ERV repeat transcription by, for example, low dose DNMT inhibition with azacytidine (DNMTi) can break immune tolerance of cancer cells and revealed a novel quality for ‘epigenetic therapy’ [11, 47]. Collectively, these insights demonstrate separate functions for the dysregulation of distinct repeat subclasses in either the progression or attenuation of human solid tumors. For hematopoietic malignancies, such as AML, only very few studies on the expression/dysregulation of repeat elements were done [13, 14].

### Expression analysis of distinct repeat elements in AML

We performed an integrative bioinformatic analysis of repeat element expression/dysregulation in human AML. First, we used primary leukemic cells from AML patients (Uniklinik Freiburg) and processed non-poly(A) selected total RNA for Hiseq RNA sequencing. Non-poly(A) selected RNA libraries maximize the read coverage and will also include satellite repeat transcripts and truncated LTR/ERV transcripts which would lack poly(A) tails. We find that several satellite repeats (e.g. ALRalpha, LSAU and HSATII/III) and LTR/ERV repeats (e.g. distinct LTR/ERV1 subtypes) are derepressed in the AML patient (Uniklinik Freiburg) samples as compared to healthy CD34 + control cells (see Fig. 1c/d and Additional file 3: Figure S3).

We then extended this analysis to poly(A) selected RNA sequencing data sets from the Blueprint and TCGA consortia that together comprised 112 AML patient samples. For satellite repeats, we can identify a number of subtypes, including, for example, SATR2 (minisatellite present on human chromosomes 3, 5, 16, 19 and 22), MSR1 (minisatellite present on human chromosome 19) and GSATII (centromeric gamma satellite) that display elevated transcript levels in individual patients (see Additional file 5: Figure S5). For LTR/ERV repeats, several transcripts, such as LTR12C ERV1, LTR12E ERV1 and LTR18C ERVL transcripts also appear derepressed in some patient samples (see Additional file 6: Figure S6). These repeat subtypes are different from a subset of 14 transposable elements (e.g. ALUJo, MER11A, LTR14A and others) that were recently described as being prognostic biomarkers in TCGA AML patient samples and where expression of LTR14A, LTR45B, MER77 and Tigger9b are hazardous covariates that correlate with increased AML risk [14]. The distinct repeat subtypes identified in our study and in previous work [13, 14] appear to be the most susceptible repeat elements that are found to have altered expression in AML.

The expression of satellite repeats is generally very low (only around 0.2% of all repeat transcripts), whereas LTR/ERV repeat transcripts reach 9–12% (see Figs. 1a and 2b). LTR12C seems to be an exceptionally ‘hot’ repeat element, since it is also very highly expressed in several cancer cell lines [39] and appears particularly responsive to derepression by a combination treatment with vitamin C and decitabine (DNMTi) in these cancer cells [39] or in mobilized healthy human CD34 + cells (PhD thesis Zoe [50]. DNMTi and HDACi inhibitors have recently been shown to activate cryptic transcription start sites in the LTR12 ERV9 repeat family in a lung cancer cell line [6]. While LINE and SINE/ALU repeat transcripts display the highest expression of repeat elements (each at around 30–40%), we have not found appreciable dysregulation of distinct LINE or SINE/ALU elements in AML patient samples (see Additional file 4: Figure S4 and data not shown). A recent study has demonstrated that MDA5, a cytosolic dsRNA sensor that induces anti-viral immune response and inflammation is specifically stimulated by inverted ALU duplex RNA repeats [1].

### Repeat to gene expression ratio as an instructive signature for repeat element dysregulation

Whereas we do not exclude that there is dysregulation of some satellite and LTR/ERV repeat elements in human AML, we have observed considerable heterogeneity in the expression of all repeat subclasses and significant inter-patient variation (see Fig. 1c/d and Additional file 5: Figure S5 and Additional file 6: Figure S6). We therefore derived another method that is not restricted to the analysis of distinct repeat subclasses or individual repeat elements and which can quantify the expression of all repeat transcripts in relation to the expression of all gene and non-repeat transcripts (as described above and also see "Methods" section). This approach therefore establishes a repeat to gene expression ratio (R/G ratio), which can be used as an instructive signature for repeat element expression and its possible adaptation to different physiological or pathological settings.

Indeed, R/G ratios differ significantly between hematopoietic stem cells (HSC), multipotent progenitor cells (MPP) and common myeloid progenitor cells (CMP), as they were subtyped in the Blueprint data sets. While HSC have a high R/G ratio, there is a progressive decrease in repeat expression in MMP and in CMP (see Fig. 2a). These data suggest that the R/G ratio model can distinguish subpopulations of hematopoietic cells at various stages of their differentiation and that HSC display the highest level of repeat element expression. Similar to embryonic stem cells [18, 31], HSC may have a more accessible chromatin structure, possibly allowing for less restricted transcriptional activity across the entire genome.

The R/G ratio model also discriminated M1, M2 and M4 AML subtypes (Uniklinik Freiburg), such that overall repeat expression was lowest in the more differentiated leukemic cells of the M4 AML patient samples (see Fig. 1b). Importantly, we could use R/G ratios to sub-stratify AML patient samples into two distinct subgroups with either a low or high overall repeat expression (see Fig. 3a/c). This R/G ratio filtered sub-stratification correlated with survival probability, such that ‘low-repeat’ AML patients have a poorer prognosis as compared to ‘high-repeat’ AML patients (see Fig. 5a). Since the R/G ratio model only relies on the relative expression of repeat vs. non-repeat (gene) transcripts, it is independent of the FAB classification or other cytogenetic (chromosomal aberrations) and gene mutation based variables (Fig. 5b). We therefore propose the R/G ratio model as an instructive signature that can be used to distinguish hematopoietic stem/progenitor cells from more differentiated cells and as a novel biomarker for the analysis of repeat element dysregulation in human AML.

### The R/G ratio model can identify low-risk and high-risk AML patients

Gene expression ontology annotations and IPA analyses show that ‘low-repeat’ and ‘high-repeat’ AML patient samples display a similar activation of pattern recognition pathways and interferon response as compared to healthy CD34 + control cells (see Additional file 9: Figure S9), but differ in the dysregulation of other gene expression pathways.

In the ‘low-repeat’ AML patient samples, there appears to be an uncoupling of the sensory (activation of immune response) and the operational (suppression of infection and inflammation pathways) components of an anti-tumor immune response (see Fig. 4a), possibly indicating that leukemic cells in the ‘low-repeat’ AML patients are more likely to be immuno-tolerant. In addition, down-regulation of inflammation pathways entails reduced growth factor and cytokine signaling (see Fig. 4b), resulting in less differentiation. Leukemic stem cells have been associated with chemotherapy resistance and higher rates of relapse and show broader down-regulation of repeat elements as compared to leukemic blast cells [13]. Drug-tolerant cancer cells also are characterized by profound repression of LINE-1 elements [27].

‘High-repeat’ AML patient samples, on the other hand, display suppression of cancer-promoting pathways and activation of a death/apoptosis pathway (see Fig. 4a/b). In addition, we found a statistically significant correlation (*p*-value of 0.0048 (n = 8), Wilcoxon rank-sum test) for *RUNX1* mutations (one of the top recurring gene mutations in AML) in the ‘high-repeat’ AML patients that was not as apparent for other gene mutations (e.g. *DNMT3A, NMP1* or *FLT3*) or for *TP53* (*p*-value of 0.04 (n = 6), Wilcoxon rank-sum test) (Additional file 10: Figure S10). RUNX1 has been found to associate with the H3K9 KMT SUV39H1 and with HDAC to repress transcription [46]. A recent systematic profiling of chromatin signatures and gene mutations in AML patient samples has identified two distinct subtypes, in which a *RUNX1*-mutant subgroup appears to represent more late-stage leukemic cells with a better prognosis [65].

## Conclusions

**Fig. 7:**
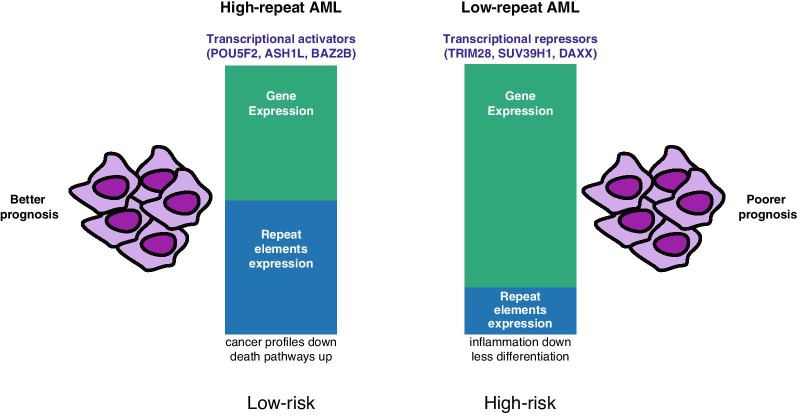
Summary model for repeat expression in low-risk and high-risk AML patients. Schematic representation of the relative gene expression (green) versus repeat element expression (blue) in High-repeat AML patients (left) and Low-repeat AML patients (right). High-repeat AML patients have a higher survival probability (low-risk AML group), whereas Low-repeat AML patients have a lower survival probability (high-risk AML group)

In summary, our integrative bioinformatic analysis of repeat element dysregulation in human AML revealed that some satellite repeats and distinct LTR/ERV repeat subtypes are derepressed in a sizeable fraction of AML patient samples, although there is considerable heterogeneity among individual repeat elements and significant inter-patient variation. This inter-patient variation can, at least in part, be restructured by a new bioinformatic approach that quantifies the expression of all repeat transcripts versus the expression of all non-repeat transcripts. We show that this R/G ratio model can be used as a biomarker to sub-stratify AML patients into low-risk and high-risk subgroups (model Fig. 7). ‘Low-repeat’ AML patients have a lower survival probability (high-risk AML group), whereas ‘high-repeat’ AML patients have a higher survival probability (low-risk AML group). Our data further suggest that ‘low-repeat’ AML patients are likely to be more refractory to epigenetic therapy and may require combination treatments, such as DNMTi and HDACi plus retinoic acid or even new inhibitors that target other chromatin enzymes/factors. Attractive candidates for these additional chromatin enzymes/factors could be the SUV39H1 and G9A KMT, the TRIM28/KAP1 corepressor and the DAXX H3.3 chaperone (see Fig. 6). Although more functional work will be necessary, the results presented here illustrate that the R/G ratio model can be a valuable biomarker for AML and probably also other forms of human cancer.

## Supplementary Information

**Additional file 1**: Figure S1: Repeat elements in the human genome. (A) Ideogram of a human chromosome highlighting distinct types of repeat classes. (B) Pie chart of DNA composition of the human genome. 48% of the human genome comprise unique sequences and 52% comprise repeat sequences. The distinct repeat classes are ~22% Long Interspersed Nuclear Elements (LINE), ~13% Short Interspersed Nuclear Elements (SINE), ~9% Long Terminal Repeats (LTR/ERV), ~4% DNA transposons and ~4% Satellite repeats. (C) Schematic representation of the basic organization of distinct types of repeat elements. Shown are examples of HSATII,III, ALR, LTR/ERV, LINE, SINE(ALU) and DNA transposons. Because it is difficult to distinguish between HSATII and HSATIII, we refer to HSATII,III as the combination of (GAATG)n/(CATTC)n tandem-repeats and the diverged, ~170bp (GAATG)n sequence [60]. Full-length ERV elements comprise retroviral coding sequences (GAG, POL, ENV) and their regulatory sequences (5’ and 3’ LTR) and range in size from 6-11 kb. Solo-LTR, a product of recombination between two LTR resulting in the removal of the retroviral coding sequences, are much smaller. Importantly, the number of annotated LTR is nearly 6-fold greater than the internal retroviral coding sequence (Smit et al., n.d.), indicating that solo-LTR significantly outnumber full-length ERV. Similarly, full-length LINE elements with ORF1 and ORF2 are typically around 6 kb, but there are many degenerated and truncated LINE elements (consisting primarily of the 3’UTR) throughout the genome.

**Additional file 2**: Figure S2: DNA sequences for regulatory elements of distinct repeat classes. Shown are the consensus DNA sequences (identified by tandem repeat finder for the human genome) of the basic unit of ALR (171 bp), 5’ LTR of the LTR12C ERV (160 bp) and 5’UTR of the L1PA14 LINE element (268 bp). Predicted transcription factor binding sites and relevant transcription factors that can impart transcriptional competence to these regulatory sequences are indicated.

**Additional file 3**: Figure S3: MA plots for distinct repeat element expression in M1, M2 and M4 AML patient samples (Uniklinik Freiburg). MA-plots (mean expression versus the log2 fold change) depicting changes in repeat expression in the CD34+ controls (n=5) and the M1 (n=2), M2 (n=3) and M4 (n=3) AML subtypes. Dots above and below the dashed lines are statistically significant as confirmed by multiple testing adjustments using the BH (Benjamini-Hochberg) method. Some examples for de-repressed Satellite repeats and LTR/ERV are highlighted. Red dots represent Satellite repeats, blue dots LTR/ERV elements and orange dots LINE repeats.

**Additional file 4**: Figure S4. Heatmaps for dysregulated repeat element expression in Uniklinik Freiburg and Blueprint AML samples. (A) Top 30 statistically significant (absolute value of log2FoldChange >1; adjusted p-value <0.05) dysregulated repeat subtypes in Uniklinik Freiburg AML (AML) (n=8) versus Uniklinik Freiburg CD34+ control (n=5) samples. (B) Top 30 statistically significant (absolute value of log2FoldChange >1; adjusted p-value <0.05) dysregulated repeat subtypes in Blueprint AML (sample identifier shown by ERS number) (n=14) versus Blueprint control (HSC, MPP, CMP) (n=9) samples. In these heatmaps, the repeat subtypes are sorted and show the most up-regulated repeat subtypes at the left and the most down-regulated repeat subtypes at the right. There is no common repeat subtype that is up-regulated in these top 30 hits for the Uniklinik Freiburg or the Blueprint AML samples and only the D20S16 satellite (highlighted in red) is found to be down-regulated in both data sets.

**Additional file 5**: Figure S5: Inter-patient variation plots for expression of 12 Satellite repeat subtypes in TCGA AML samples. Inter-patient variation plots display normalized read counts of distinct Satellite repeat transcripts that are above the expression cutoff (baseMean expression > 100 normalized reads). Y-axis indicates the normalized count. Each dot represents one TCGA AML patient sample. The black horizontal line specifies the median expression of the repeat across the entire dataset including the M1 (n=35), M2 (n=35) and M4 (n=28) TCGA AML patient samples, as shown in Figure 3A.

**Additional file 6**: Figure S6: Inter-patient variation plots for expression of 12 LTR/ERV repeat subtypes in TCGA AML samples. Inter-patient variation plots display normalized read counts of distinct LTR/ERV repeat transcripts that are above the expression cutoff (baseMean expression > 100 normalized reads). Y-axis indicates the normalized count. Each dot represents one TCGA AML patient sample. The black horizontal line specifies the median expression of the repeat across the entire dataset including the M1 (n=35), M2 (n=35) and M4 (n=28) TCGA AML patient samples, as shown in Figure 3A.

**Additional file 7**: Figure S7. Coverage and distribution of repeat transcripts in Uniklinik Freiburg samples and in the Blueprint and TCGA data sets. (A) Fraction of all repeat transcripts (blue segment) versus the fraction of all protein coding transcripts (green) and other non-repeat transcripts in Uniklinik Freiburg control (n=5), Uniklinik Freiburg AML (n=8), Blueprint control (n=9), Blueprint AML (n=14) and TCGA AML (n=98) sequencing groups. (B) Fraction of transcripts for distinct repeat classes that can be detected within the repeat coverage (blue segments) shown in A. SINE/ALU transcripts are shown in yellow, Satellite transcripts in red, ERV/LTR transcripts in blue and LINE transcripts in orange.

**Additional file 8**: Figure S8: Inter-patient variation plots for expression of repeat element subclasses in ‘low-repeat’, ‘mid-repeat’ and ‘high-repeat’ AML patient samples. Inter-patient variation plots display normalized read counts in every sub-stratified Low-repeat (Low, n=15), Mid-repeat (Mid, n=89) and High-repeat (High, n=16) AML patient sample. The plots indicate that all repeat classes (Satellites, LTR/ERV, LINE and SINE/ALU elements) equally segregate with R/G ratios, as defined by the cut_interval function. Y-axis indicates the normalized count. Each dot represents one AML patient sample. The black horizontal line specifies the median R/G ratio of the repeat across the entire dataset.

**Additional file 9**: Figure S9: Activation of Toll-like receptor and interferon signaling in ‘high-repeat’ and ‘low-repeat’ AML patient samples. Ingenuity pathway analysis (IPA) performed in High-repeat AML patient samples (n=16) vs. CD34+ Blueprint samples (n=9) and in Low-repeat AML patient samples (n=15) vs. CD34+ Blueprint samples (n=9). As inputs for the IPA, the log2FoldChange values from the differential gene expression analysis were used, after filtering for a minimum expression (baseMean expression >100 normalized reads), fold-change (absolute value of log2FoldChange >1), and significance (adjusted p-value <0.05). z-score values are indicated by color code, where orange represents up-regulation of a pathway. The numbers specify the number of genes that are upregulated in each of the different pathways.

**Additional file 10**: Figure S10. Correlation between recurring gene mutations and R/G ratios in TCGA AML patient samples. (A) Box plot showing the most frequent gene mutations (x axis) in the TCGA AML cohort (n=98), as they were identified using metadata available from the TCGA study. The y-axis shows their corresponding R/G ratios. (B) Box plot correlating R/G ratios with specific gene mutations for DNMT3A (not significant, n=21), RUNX-1 (p-value 0.0048, n=8) and TP53 (p-value 0.04, n=6).

**Additional file 11**: Table 1: Identifiers and cytogenetic markers of AML patients and healthy controls (Uniklinik Freiburg). The Table shows the patient ID, gender and age and the patient karyotype. In addition, it also lists the FAB (French-American-British) and ELN (European LeukemiaNet) classification of AML for each patient. Percentages of leukemic blast cells in bone marrow (BM) and peripheral blood (PB) for each AML patient are also indicated.

**Additional file 12**: Table 2: Listing of chromatin enzymes/factors that were interrogated for expression changes in AML patient samples displaying different R/G ratios.

## Data Availability

The datasets generated and/or analysed during the current study are available in the GSE175701 repository, https://www.ncbi.nlm.nih.gov/geo/query/acc.cgi?acc=GSE175701. Blueprint data sets are available in the https://europepmc.org/articles/PMC6363099 repository. TCGA data sets are available in the https://portal.gdc.cancer.gov/projects/TCGA-LAML repository.

## Abbreviations

AML: Acute myeloid leukemia
CMP: Common myeloid progenitor cells
DNMT: DNA methyltransferase inhibitor
ERV: Endogenous retrovirus
HDAC: Histone deacetylase inhibitors
HSC: Hematopoietic stem cells
KMT: Histone lysine methyltransferase
MPP: Multipotent progenitor cells
MSR: Major satellite repeat
LINE: Long interspersed nuclear elements
LTR: Long terminal repeat
PCC: Pearson correlation coefficient
R/G ratio: Repeat to gene expression ratio
SEM: Standard error of the mean
SINE: Short interspersed nuclear elements
TCGA: The cancer genome atlas
