# Supplementary material for: Repeat to gene expression ratios in leukemic blast cells can stratify risk prediction in acute myeloid leukemia
Source: BMC Med Genomics. 2021 Jun 26;14:166. doi: 10.1186/s12920-021-01003-z (PMC8234671; doi:10.1186/s12920-021-01003-z)
Supplement: Supplementary file 1 — Additional file 1: Figure S1: Repeat elements in the human genome. (A) Ideogram of a human chromosome highlighting distinct types of repeat classes. (B) Pie chart of DNA composition of the human genome. 48% of the human genome comprise unique sequences and 52% comprise repeat sequences. The distinct repeat classes are ~22% Long Interspersed Nuclear Elements (LINE), ~13% Short Interspersed Nuclear Elements (SINE), ~9% Long Terminal Repeats (LTR/ERV), ~4% DNA transposons and ~4% Satellite repeats. (C) Schematic representation of the basic organization of distinct types of repeat elements. Shown are examples of HSATII,III, ALR, LTR/ERV, LINE, SINE(ALU) and DNA transposons. Because it is difficult to distinguish between HSATII and HSATIII, we refer to HSATII,III as the combination of (GAATG)n/(CATTC)n tandem-repeats and the diverged, ~170bp (GAATG)n sequence [60]. Full-length ERV elements comprise retroviral coding sequences (GAG, POL, ENV) and their regulatory sequences (5’ and 3’ LTR) and range in size from 6-11 kb. Solo-LTR, a product of recombination between two LTR resulting in the removal of the retroviral coding sequences, are much smaller. Importantly, the number of annotated LTR is nearly 6-fold greater than the internal retroviral coding sequence (Smit et al., n.d.), indicating that solo-LTR significantly outnumber full-length ERV. Similarly, full-length LINE elements with ORF1 and ORF2 are typically around 6 kb, but there are many degenerated and truncated LINE elements (consisting primarily of the 3’UTR) throughout the genome. [file 12920_2021_1003_MOESM1_ESM.pdf]

Patient ID	Gender /Age	Karyotype	FAB	ELN 2010 risk	% blasts in BM/ PB
**TM10**	m/70	91,XXYY,del(2)(q32q37),der(5)t(?;5)(?;p14)del(5)(q14q35),+13,+13 3, -17,-21,-21 [1] / 46,XY [8]	M1	adverse	78 / 82 (bone marrow)
**019**	m/66	46,XY [21]	M1	int-1	76 /78
**TM03**	f/82	46,XX [20]	M2	int-1	n.a. / 72
**TM11**	f/68	46,XX [20]	M2	int-1	n.a. / 77
**005**	f/60	47,XX,t(8;21)(q22;q22),+mar[20]	M2	favorable	45 / 40 *
**TM04**	m/47	46,XY,del(11)(q14q25) [19] / 46,XY,t(3;15)(p11;q21),del(11)(q14q25) [2]	M4	int-2	37 / 31 *
**010**	f/47	46,XX,del(7)(q31q36),inv(16)(p13.1q22)[2] / 47,XX,del(7)(q31q36),+8,inv(16)(p13.1q22) [10] / 47,XX,del(7)(q31q36),inv(16)(p13.1q22),+22 [6] / 50,XX,+6,+8,+13,inv(16)(p13.1q22),+22 [2] / 46,XX [1]	M4	favorable	25 / 22 *
**016**	m/74	46,XY,del(20)(q11q13)[20]	M4	int-2	90 / 90
**CD34_1**	m				
**CD34_2**	m				
**CD34_3**	m				
**CD34_5**	f				
**CD34_8**	f				

* enrichment of peripheral blood blasts

Supplemental Table 1
