# Supplementary material for: Repeat to gene expression ratios in leukemic blast cells can stratify risk prediction in acute myeloid leukemia
Source: BMC Med Genomics. 2021 Jun 26;14:166. doi: 10.1186/s12920-021-01003-z (PMC8234671; doi:10.1186/s12920-021-01003-z)
Supplement: Supplementary file 2 — Additional file 2: Figure S2: DNA sequences for regulatory elements of distinct repeat classes. Shown are the consensus DNA sequences (identified by tandem repeat finder for the human genome) of the basic unit of ALR (171 bp), 5’ LTR of the LTR12C ERV (160 bp) and 5’UTR of the L1PA14 LINE element (268 bp). Predicted transcription factor binding sites and relevant transcription factors that can impart transcriptional competence to these regulatory sequences are indicated. [file 12920_2021_1003_MOESM2_ESM.pdf]

Chromatin modifying enzymes: KMT (SET-domain)
ASH1L
EHMT1/GLP<br>EHMT2/G9A
EZH1<br>EZH2
MLL1/2/3 (KMT2A, KMT2D, KMT2C)<br>NSD1/KMT3B<br>SMYD1/KMT3D<br>NSD2/WHSC1/KMT3F<br>NSD3/KMT3G
SETD1A<br>SETD2<br>SETD5
SETDB1
SUV39H1<br>SUV39H2
SUV420H1<br>SUV420H2

Chromatin modifying enzymes: KMT (non-SET-domain)
DOT1L

Chromatin modifying enzymes: KDM
KDM2A<br>KDM2B<br>KDM3A<br>KDM3B<br>KDM4A<br>KDM4B<br>KDM4C<br>KDM5A/JARID1A<br>KDM5C/JARID1B<br>KDM6A/UTX<br>KDM6B<br>KDM7C/PHF2
PHF8 (88)

Chromatin modifying enzymes: KMT (PR/SET-domain)
PRDM2<br>PRDM9<br>PRDM3<br>PRDM16

Chromatin modifying enzymes: Kinase
AURKB
ATM
JAK2
PIM1
BRCA1

Chromatin modifying enzymes: HAT
p300/CBP (EP300)
KAT2A<br>KAT2B<br>MOZ/KAT6A<br>CREBBP/KAT3A
NCOA1<br>NCOA3
PBRM1

Chromatin modifying enzymes: HDAC
HDAC1<br>HDAC2<br>HDAC3<br>HDAC4<br>HDAC5<br>HDAC6<br>HDAC9
SIRT1

DNA methylation
DNMT1<br>DNMT3A<br>DNMT3B
MBD1<br>MBD2
MECP2
TET1<br>TET2<br>TET3

Chromatin remodeling
ARID1A<br>ARID1B
ATRX
CHD3<br>CHD4
INO80<br>INO80B<br>INO80C<br>INO80D<br>INO80E
SMARCA1/SNF2L<br>SMARCA4/BRF1<br>SMARCA5 (SNF2H)<br>SMARCC1/BAF155<br>SMARCC2

Chromatin binders
BMI1
BRD1/3/4
CBX2
DAXX
HMGA1<br>HMGA2
HP1-alpha/CBX5<br>HP1-beta/CBX1<br>HP1-gamma/CBX3
ING1<br>ING4
SSRP1
TRIM28
TRIM33

Chromatin assembly proteins
CHAF1A
CHAF1B

Supplemental Table 2
