# Supplementary figures and images for: Repeat to gene expression ratios in leukemic blast cells can stratify risk prediction in acute myeloid leukemia
Source: BMC Med Genomics. 2021 Jun 26;14:166. doi: 10.1186/s12920-021-01003-z (PMC8234671; doi:10.1186/s12920-021-01003-z)

**A.** Gene Mutations vs. R/G ratios in TCGA AML samples (n=98)

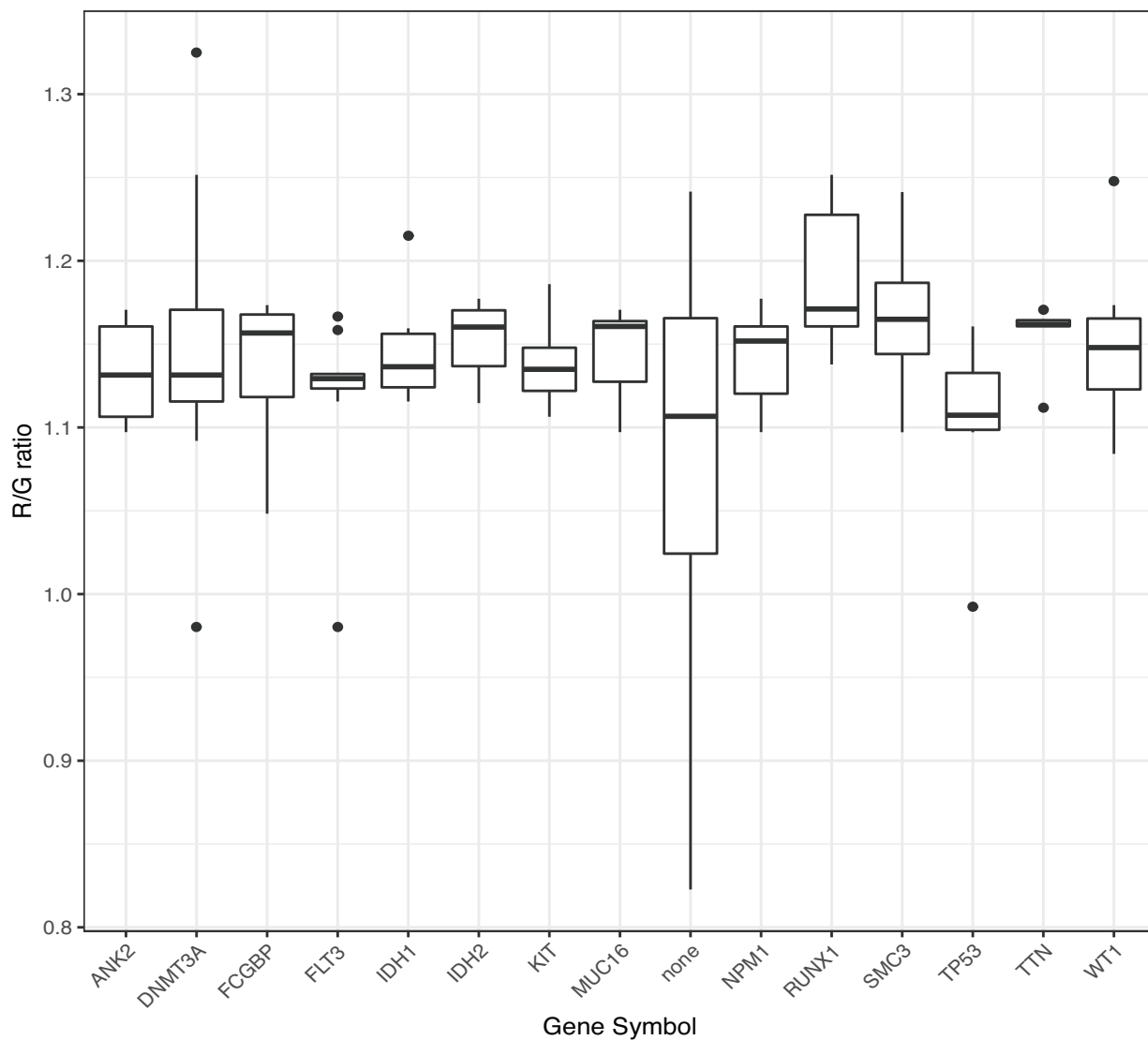

**B.**

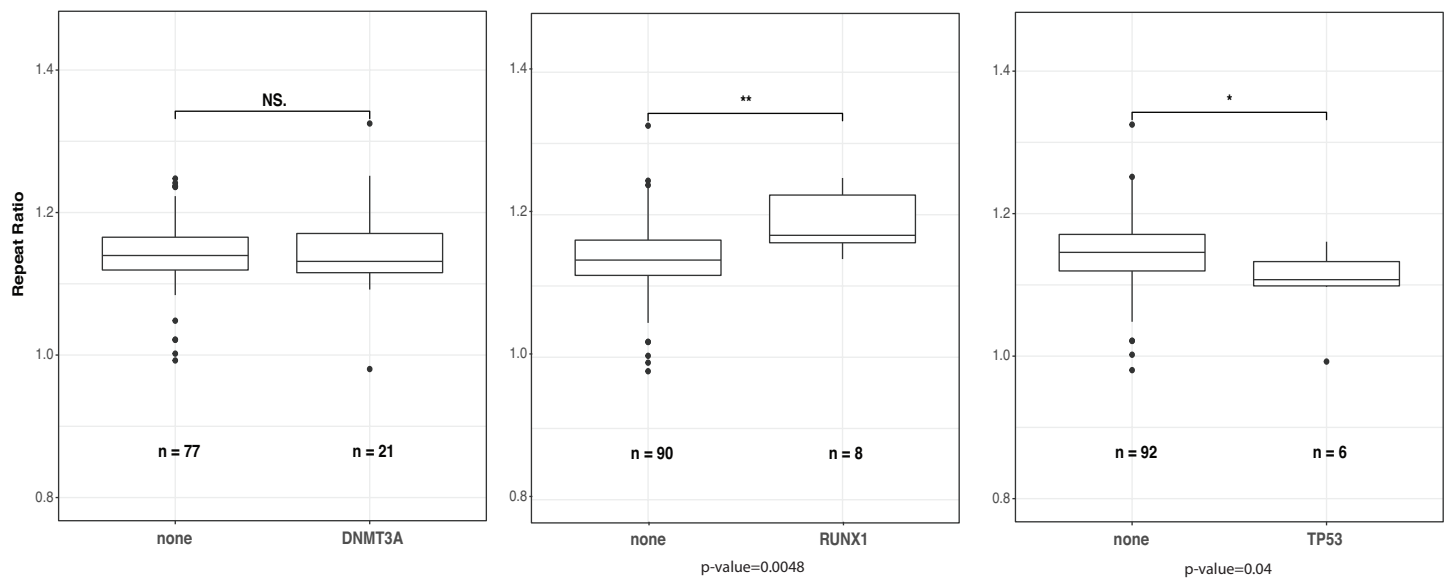

Supplement: Supplementary file 3 — Additional file 3: Figure S3: MA plots for distinct repeat element expression in M1, M2 and M4 AML patient samples (Uniklinik Freiburg). MA-plots (mean expression versus the log2 fold change) depicting changes in repeat expression in the CD34+ controls (n=5) and the M1 (n=2), M2 (n=3) and M4 (n=3) AML subtypes. Dots above and below the dashed lines are statistically significant as confirmed by multiple testing adjustments using the BH (Benjamini-Hochberg) method. Some examples for de-repressed Satellite repeats and LTR/ERV are highlighted. Red dots represent Satellite repeats, blue dots LTR/ERV elements and orange dots LINE repeats. [file 12920_2021_1003_MOESM3_ESM.pdf]

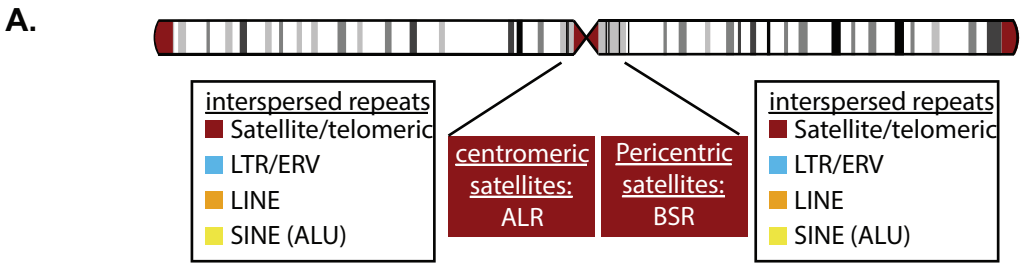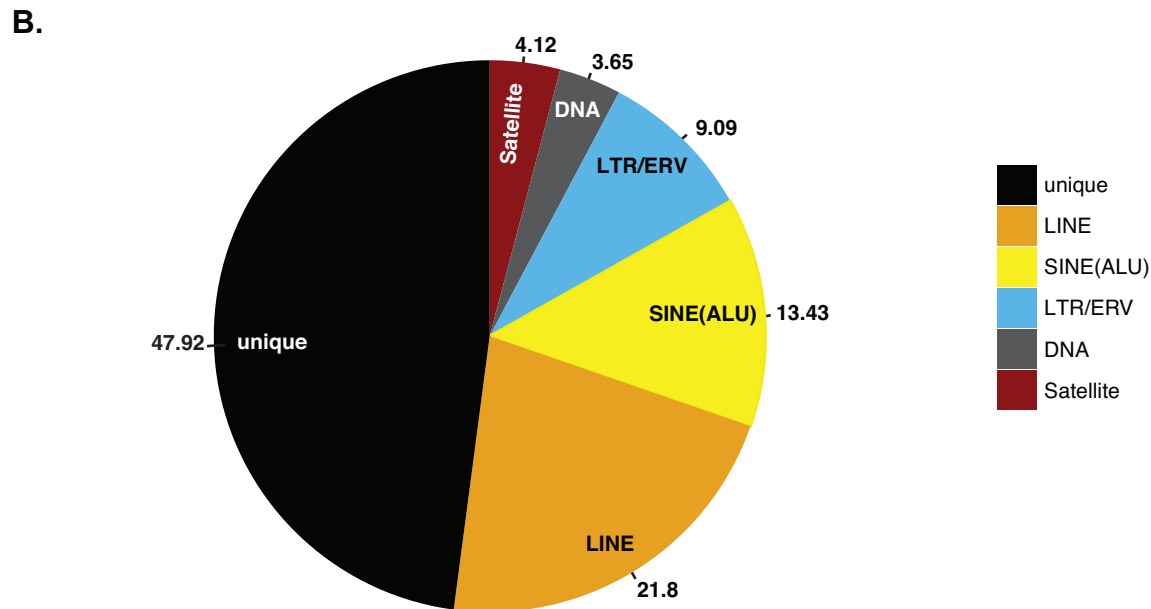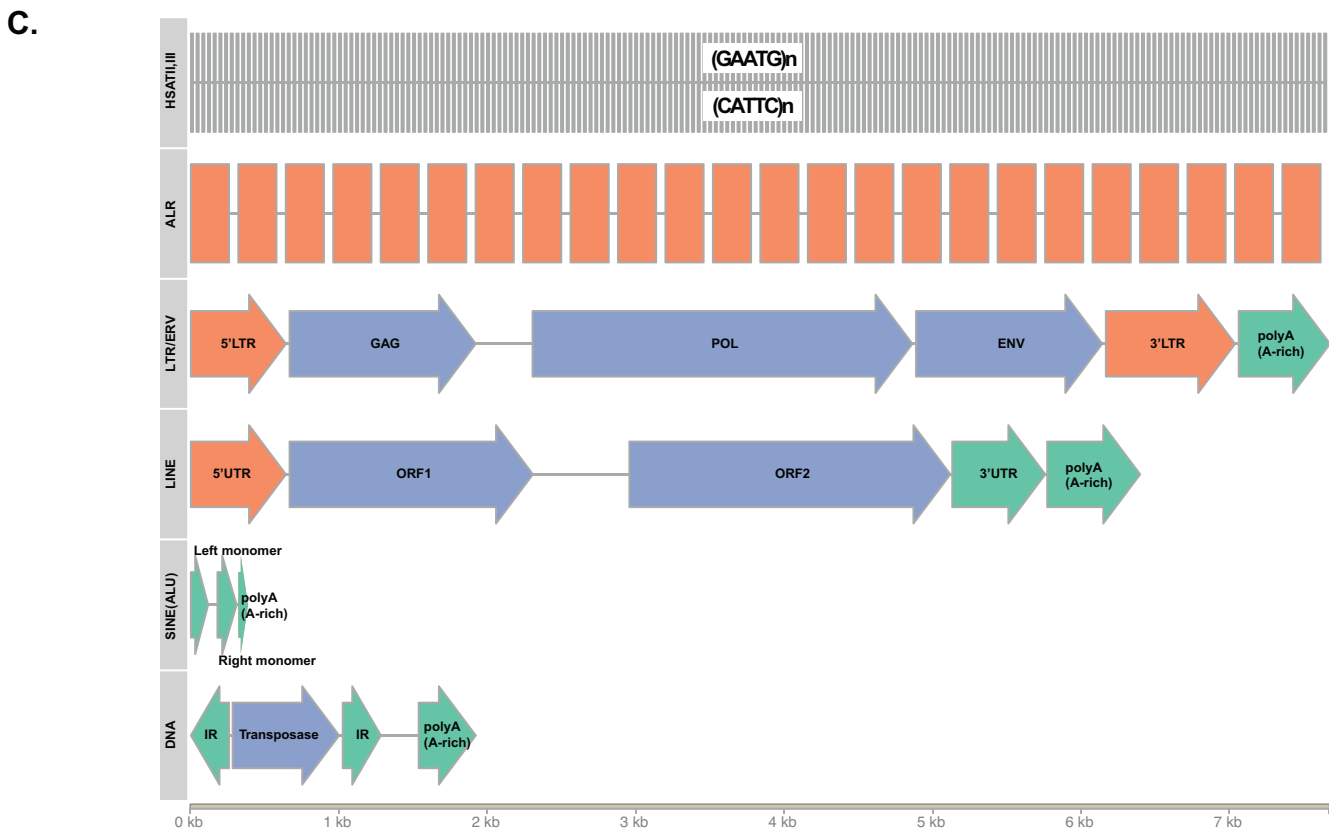

Supplementary Figure 1.

Supplement: Supplementary file 4 — Additional file 4: Figure S4. Heatmaps for dysregulated repeat element expression in Uniklinik Freiburg and Blueprint AML samples. (A) Top 30 statistically significant (absolute value of log2FoldChange >1; adjusted p-value <0.05) dysregulated repeat subtypes in Uniklinik Freiburg AML (AML) (n=8) versus Uniklinik Freiburg CD34+ control (n=5) samples. (B) Top 30 statistically significant (absolute value of log2FoldChange >1; adjusted p-value <0.05) dysregulated repeat subtypes in Blueprint AML (sample identifier shown by ERS number) (n=14) versus Blueprint control (HSC, MPP, CMP) (n=9) samples. In these heatmaps, the repeat subtypes are sorted and show the most up-regulated repeat subtypes at the left and the most down-regulated repeat subtypes at the right. There is no common repeat subtype that is up-regulated in these top 30 hits for the Uniklinik Freiburg or the Blueprint AML samples and only the D20S16 satellite (highlighted in red) is found to be down-regulated in both data sets. [file 12920_2021_1003_MOESM4_ESM.pdf]

### M1 (n=2) vs CD34 (n=5)

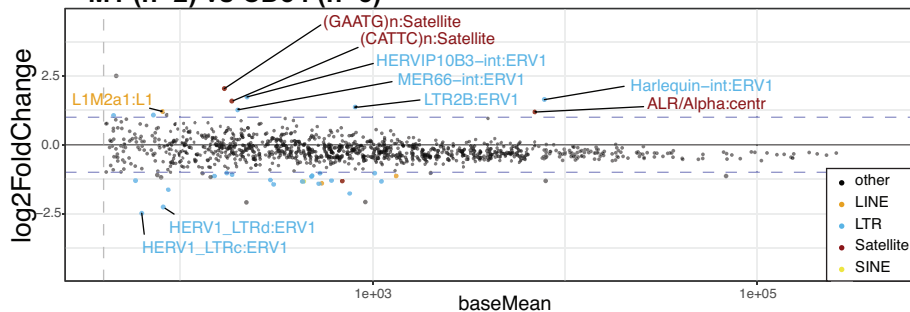

### M2 (n=3) vs CD34 (n=5)

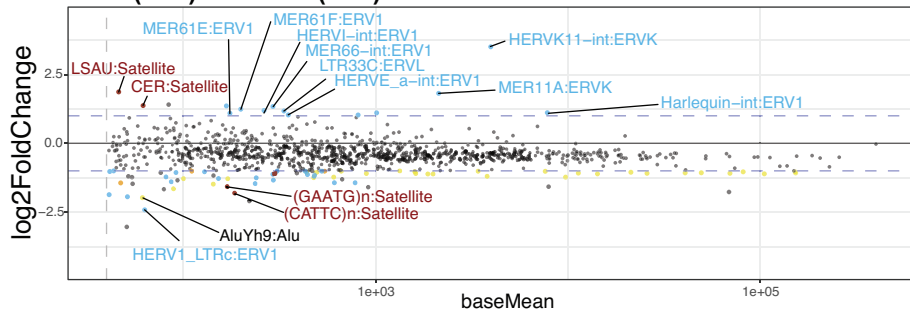

### M4 (n=3) vs CD34 (n=5)

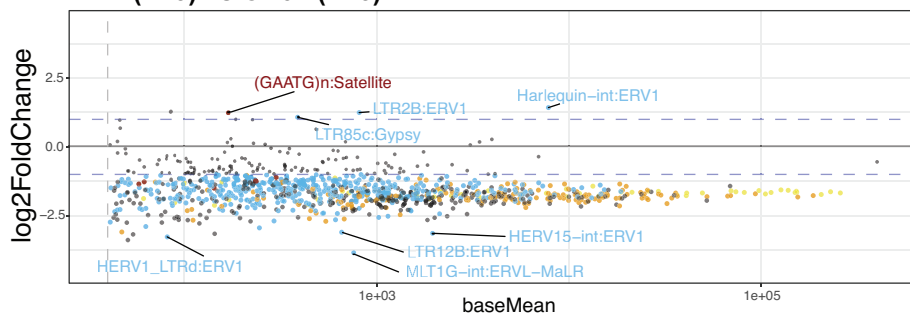

Supplement: Supplementary file 6 — Additional file 6: Figure S6: Inter-patient variation plots for expression of 12 LTR/ERV repeat subtypes in TCGA AML samples. Inter-patient variation plots display normalized read counts of distinct LTR/ERV repeat transcripts that are above the expression cutoff (baseMean expression > 100 normalized reads). Y-axis indicates the normalized count. Each dot represents one TCGA AML patient sample. The black horizontal line specifies the median expression of the repeat across the entire dataset including the M1 (n=35), M2 (n=35) and M4 (n=28) TCGA AML patient samples, as shown in Figure 3A. [file 12920_2021_1003_MOESM6_ESM.pdf]

22 Satellite types  $\xrightarrow{\text{expression cutoff}}$  15 Satellite types

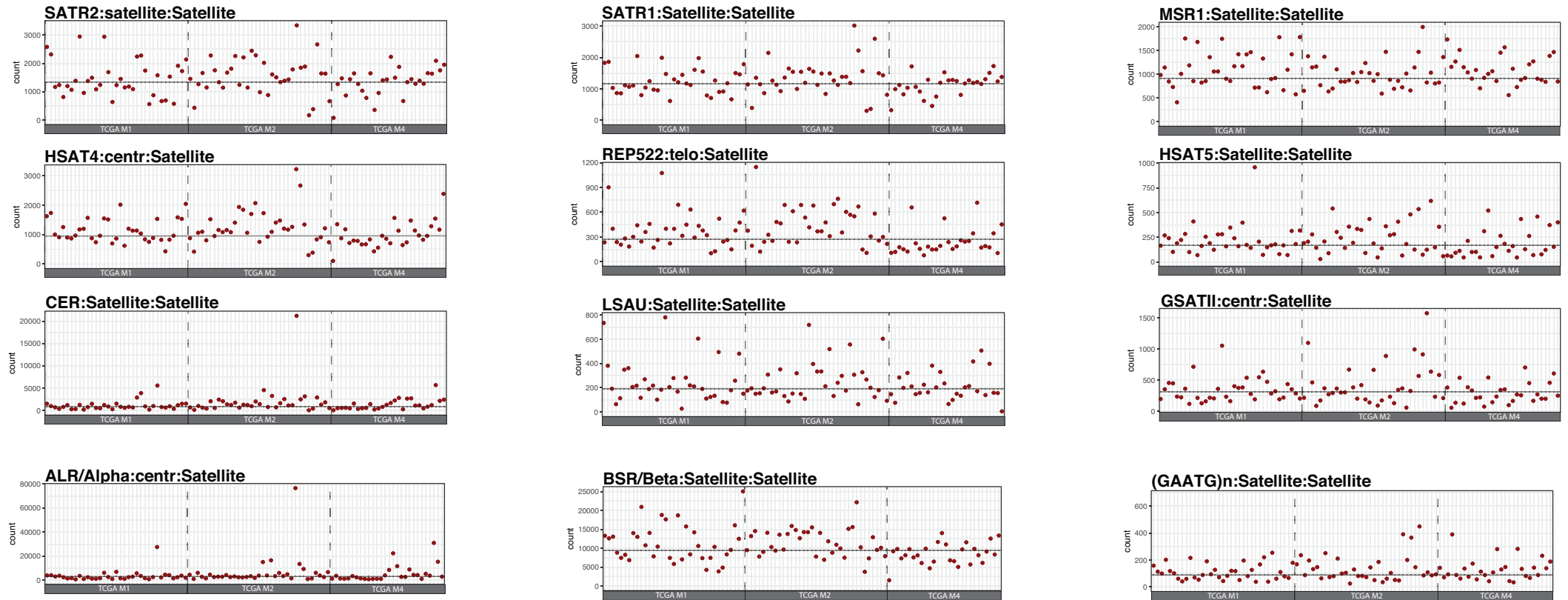

Supplementary Figure 5.

Supplement: Supplementary file 8 — Additional file 8: Figure S8: Inter-patient variation plots for expression of repeat element subclasses in ‘low-repeat’, ‘mid-repeat’ and ‘high-repeat’ AML patient samples. Inter-patient variation plots display normalized read counts in every sub-stratified Low-repeat (Low, n=15), Mid-repeat (Mid, n=89) and High-repeat (High, n=16) AML patient sample. The plots indicate that all repeat classes (Satellites, LTR/ERV, LINE and SINE/ALU elements) equally segregate with R/G ratios, as defined by the cut_interval function. Y-axis indicates the normalized count. Each dot represents one AML patient sample. The black horizontal line specifies the median R/G ratio of the repeat across the entire dataset. [file 12920_2021_1003_MOESM8_ESM.pdf]

578 LTR/ERV types  $\xrightarrow{\text{expression cutoff}}$  549 LTR/ERV types

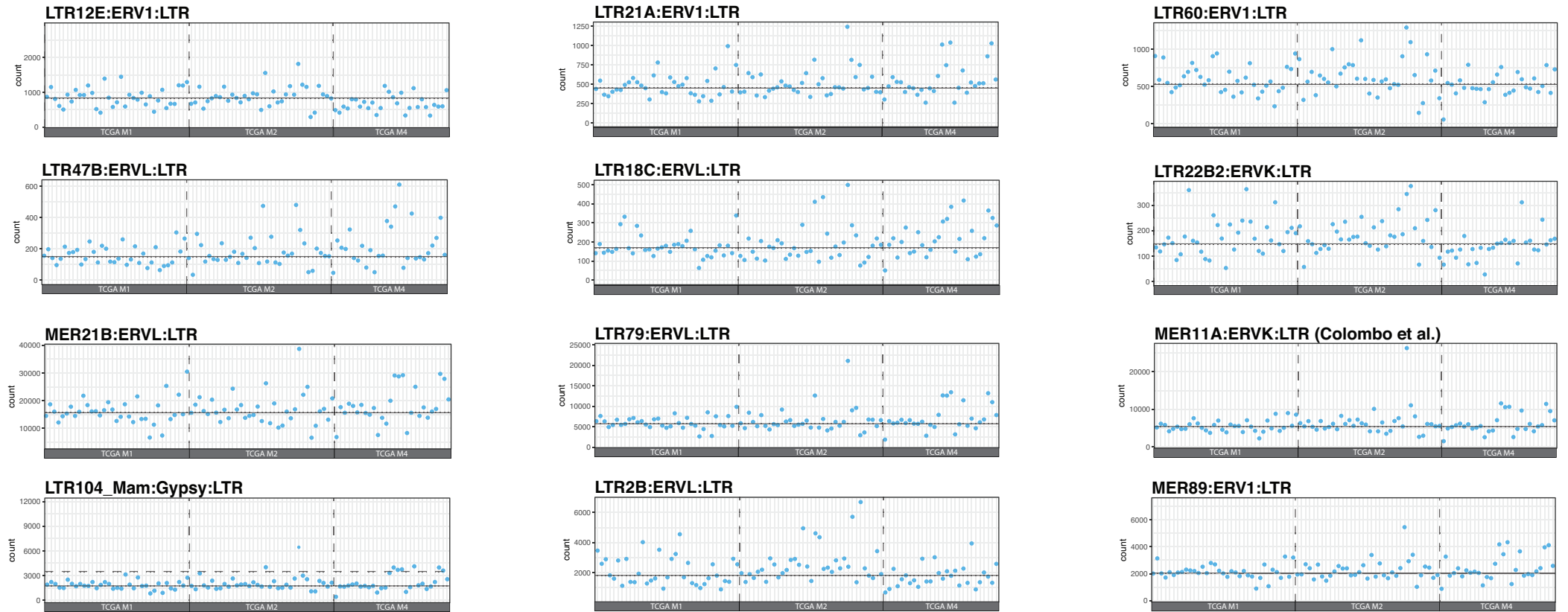

Supplementary Figure 6.

Supplement: Supplementary file 9 — Additional file 9: Figure S9: Activation of Toll-like receptor and interferon signaling in ‘high-repeat’ and ‘low-repeat’ AML patient samples. Ingenuity pathway analysis (IPA) performed in High-repeat AML patient samples (n=16) vs. CD34+ Blueprint samples (n=9) and in Low-repeat AML patient samples (n=15) vs. CD34+ Blueprint samples (n=9). As inputs for the IPA, the log2FoldChange values from the differential gene expression analysis were used, after filtering for a minimum expression (baseMean expression >100 normalized reads), fold-change (absolute value of log2FoldChange >1), and significance (adjusted p-value <0.05). z-score values are indicated by color code, where orange represents up-regulation of a pathway. The numbers specify the number of genes that are upregulated in each of the different pathways. [file 12920_2021_1003_MOESM9_ESM.pdf]

**A.**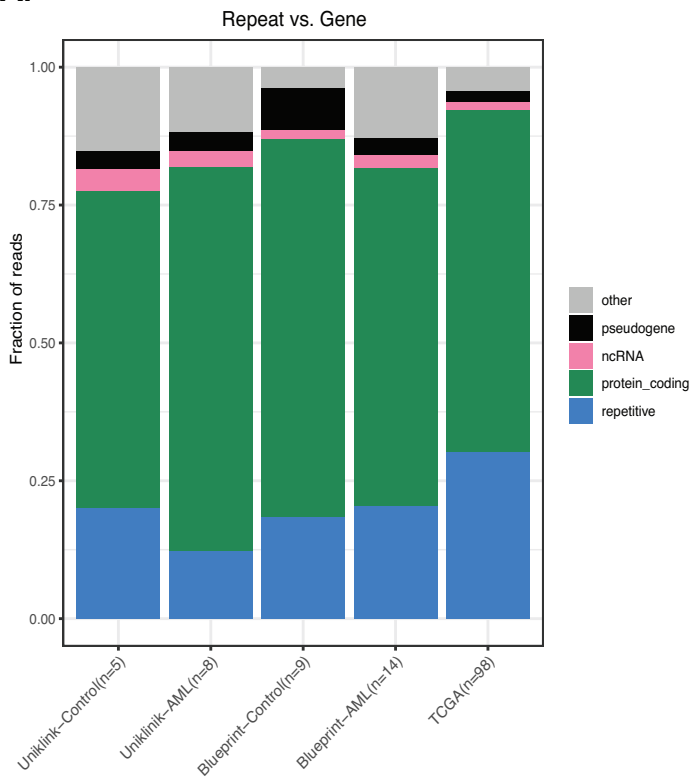**B.**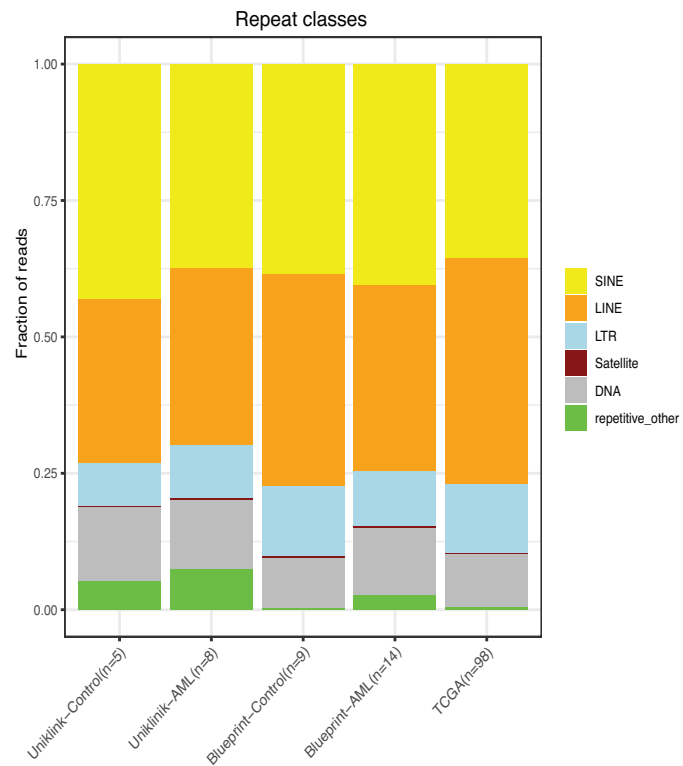

Supplement: Supplementary file 10 — Additional file 10: Figure S10. Correlation between recurring gene mutations and R/G ratios in TCGA AML patient samples. (A) Box plot showing the most frequent gene mutations (x axis) in the TCGA AML cohort (n=98), as they were identified using metadata available from the TCGA study. The y-axis shows their corresponding R/G ratios. (B) Box plot correlating R/G ratios with specific gene mutations for DNMT3A (not significant, n=21), RUNX-1 (p-value 0.0048, n=8) and TP53 (p-value 0.04, n=6). [file 12920_2021_1003_MOESM10_ESM.pdf]

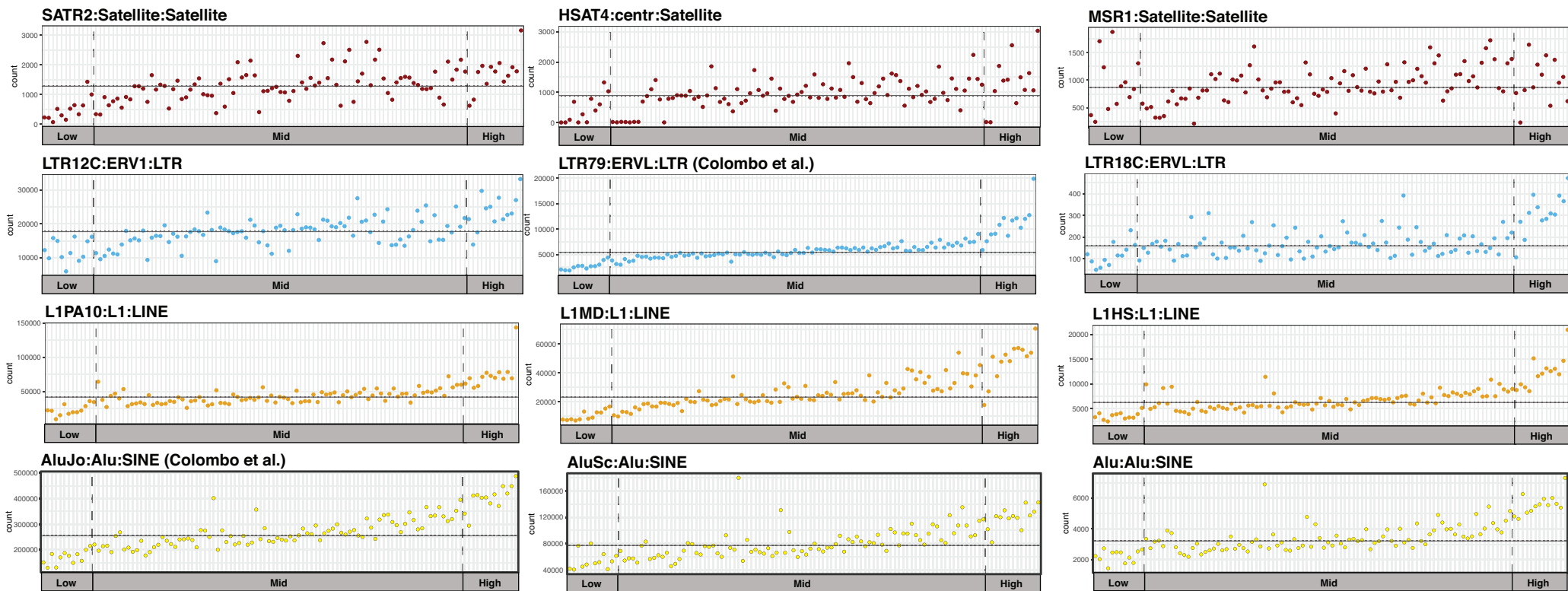

Supplementary Figure 8.

Supplement: Supplementary file 11 — Additional file 11: Table 1: Identifiers and cytogenetic markers of AML patients and healthy controls (Uniklinik Freiburg). The Table shows the patient ID, gender and age and the patient karyotype. In addition, it also lists the FAB (French-American-British) and ELN (European LeukemiaNet) classification of AML for each patient. Percentages of leukemic blast cells in bone marrow (BM) and peripheral blood (PB) for each AML patient are also indicated. [file 12920_2021_1003_MOESM11_ESM.pdf]

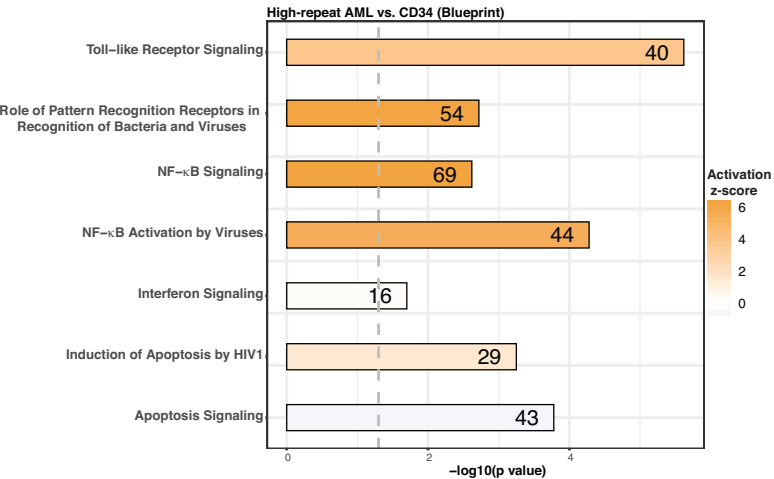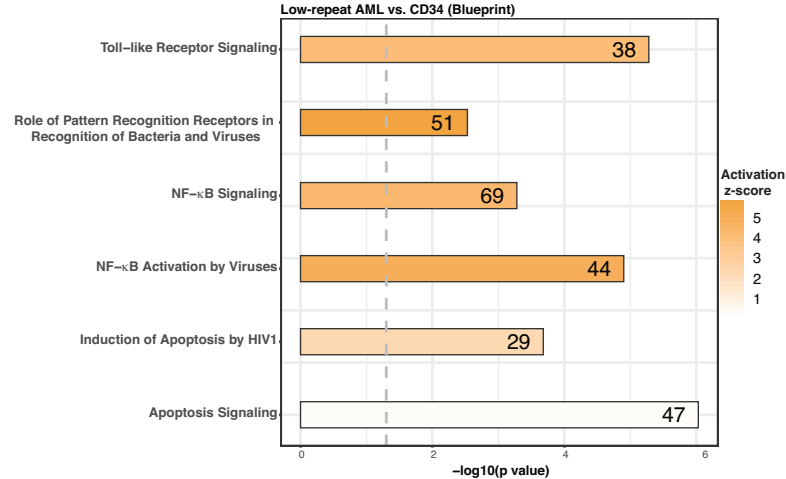

**Supplementary Figure 9.**

Supplement: Supplementary file 12 — Additional file 12: Table 2: Listing of chromatin enzymes/factors that were interrogated for expression changes in AML patient samples displaying different R/G ratios. [file 12920_2021_1003_MOESM12_ESM.pdf]
