# Supplementary material for: Repeat to gene expression ratios in leukemic blast cells can stratify risk prediction in acute myeloid leukemia
Source: BMC Med Genomics. 2021 Jun 26;14:166. doi: 10.1186/s12920-021-01003-z (PMC8234671; doi:10.1186/s12920-021-01003-z)
Supplement: Supplementary file 5 — Additional file 5: Figure S5: Inter-patient variation plots for expression of 12 Satellite repeat subtypes in TCGA AML samples. Inter-patient variation plots display normalized read counts of distinct Satellite repeat transcripts that are above the expression cutoff (baseMean expression > 100 normalized reads). Y-axis indicates the normalized count. Each dot represents one TCGA AML patient sample. The black horizontal line specifies the median expression of the repeat across the entire dataset including the M1 (n=35), M2 (n=35) and M4 (n=28) TCGA AML patient samples, as shown in Figure 3A. [file 12920_2021_1003_MOESM5_ESM.pdf]

alpha-like satellites

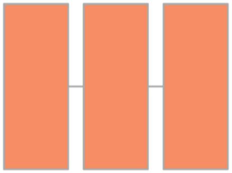

ALR (171bp, 38% GC)

```
                                C/EBPβ    FOXP3
aattctcagtaacttccttgtgttgtgtgt
ttaagagtcattgaaggaacacaacacaca

attcaactcacagagttgaacgatccttta
taagttgagtgtctcaacttgctaggaaat
                                          GR
cacagagcagacttgaaacactcttttttgt
gtgtctcgtctgaactttgtgagaaaaaca
  GR-β
ggaatttgcaagtggagatttcagccgctt
ccttaaacgttcacctctaaagtcggcgaa
   ERα    YY1          GR-α     STAT4
tgaggtcaatggtagaataggaaatatctt
actccagttaccatcttatcctttatagaa
 GR-α
cctatagaaactagacagaat
ggatatctttgatctgtctta
```

LTR/ERV repeats

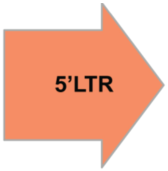

LTR12C repeat* (160bp, 45% GC)

```
tctggtggggacttggagaacctttatgtc
agaccacccctgaacctcttggaaatacag
            C/EBPβ
tagctaagggattgtaaatacaccaatcag
atcgattccctaacatttatgtggttagtc
                                C/EBPβ
cactctgtatctagctcaaggtttgtaaac
gtgagacatagatcgagttccaaacatttg
                       GR-a
acaccaatcagcaccctgtgtctagctcag
tgtggttagtcgtgggacacagatcgagtc
   C/EBPβ
ggtttgtaaatgcaccaatcgacactctgt
ccaaacatttacgtggttagctgtgagaca

atctagctaa
tagatcgatt
```

LINE repeats

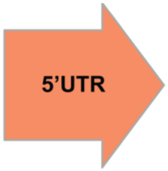

L1PA14 5UTR repeat* (268bp, 64% GC)

```
acctccctgggacggagctcccagagggag
tggagggaccctgcctcgagggtctccctc
        YY1   C/EBPβ    TCF-4E
gggcgggccgccatctttgctgtttgggcg
cccgcccggcggtagaaacgacaaacccgc

acttagccgttccagccttcgggctttgga
tgaatcggcaaggtcggaagcccgaaacct

gagtccgagccgaccgggggcggaagggtc
ctcaggctcggctggcccccgccttcccag

ccccagcacagcacagctgctctacgaaaa
ggggtcgtgtcgtgtcgacgagatgctttt
                      TFIID
cgtggccagactgctttttttaagcgggtcc
gcaccggtctgacgaaaaaattcgcccagg
     YY1
ccgatcccattcctcctcactgggcgggac
ggctagggtaaggaggagtgacccgccctg

ctcccaaccggggtctccagccacccctgc
gagggttggccccagaggtcggtggggacg

cggtgttctccggccgacagagatttga
gccacaagaggccggctgtctctaaact
```

*tandem repeat finder consensus sequence

Supplementary Figure 2.
