# Supplementary material for: Repeat to gene expression ratios in leukemic blast cells can stratify risk prediction in acute myeloid leukemia
Source: BMC Med Genomics. 2021 Jun 26;14:166. doi: 10.1186/s12920-021-01003-z (PMC8234671; doi:10.1186/s12920-021-01003-z)
Supplement: Supplementary file 7 — Additional file 7: Figure S7. Coverage and distribution of repeat transcripts in Uniklinik Freiburg samples and in the Blueprint and TCGA data sets. (A) Fraction of all repeat transcripts (blue segment) versus the fraction of all protein coding transcripts (green) and other non-repeat transcripts in Uniklinik Freiburg control (n=5), Uniklinik Freiburg AML (n=8), Blueprint control (n=9), Blueprint AML (n=14) and TCGA AML (n=98) sequencing groups. (B) Fraction of transcripts for distinct repeat classes that can be detected within the repeat coverage (blue segments) shown in A. SINE/ALU transcripts are shown in yellow, Satellite transcripts in red, ERV/LTR transcripts in blue and LINE transcripts in orange. [file 12920_2021_1003_MOESM7_ESM.pdf]

A.

B.

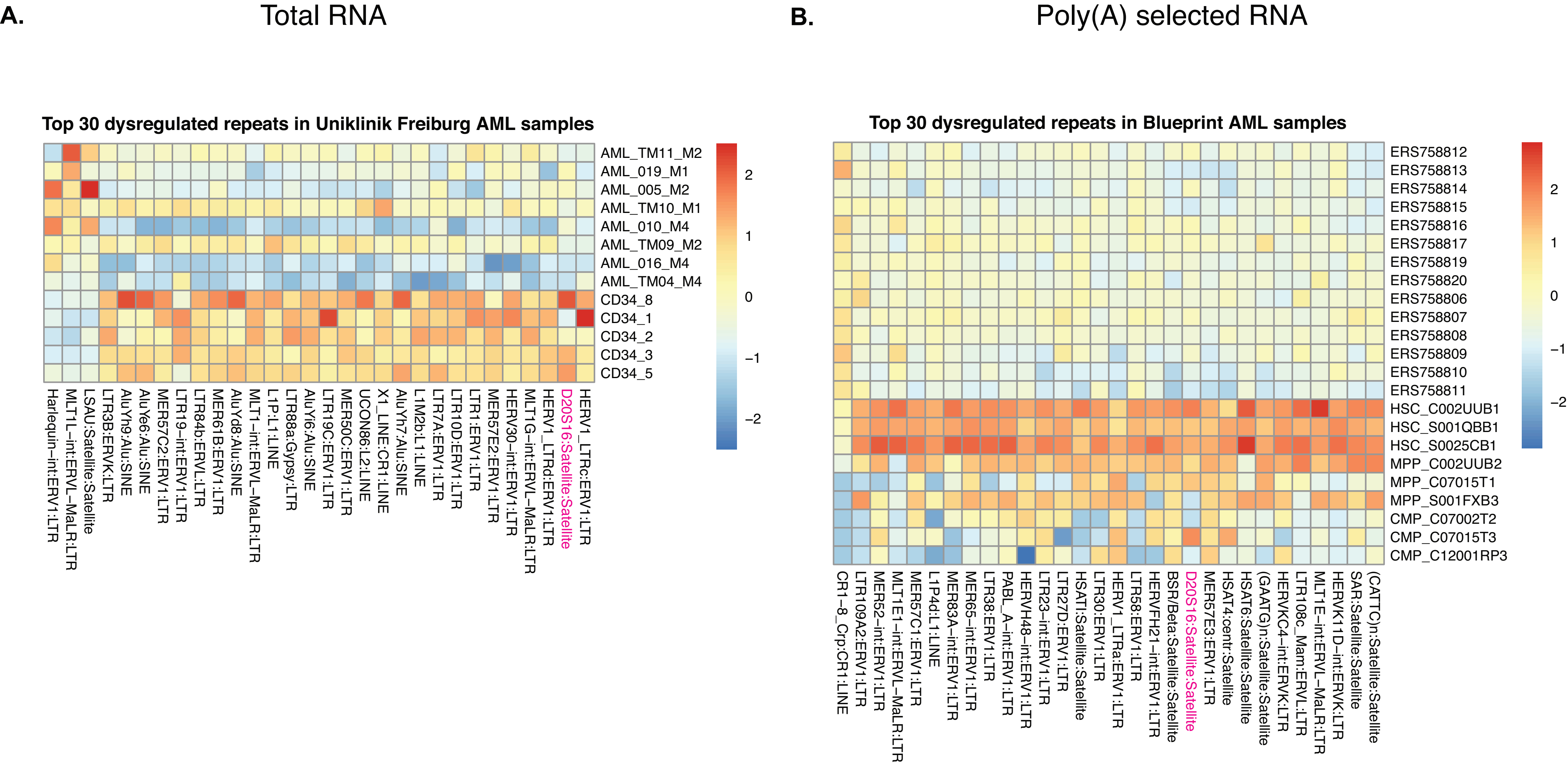

Supplementary Figure 4.
